# Synthetic Strategy Towards Heterodimetallic Half-Sandwich Complexes Based on a Symmetric Ditopic Ligand

**DOI:** 10.3389/fchem.2021.786367

**Published:** 2021-12-03

**Authors:** Lewis P. M. Green, Tasha R. Steel, Mie Riisom, Muhammad Hanif, Tilo Söhnel, Stephen M. F. Jamieson, L. James Wright, James D. Crowley, Christian G. Hartinger

**Affiliations:** ^1^ School of Chemical Sciences, University of Auckland, Auckland, New Zealand; ^2^ Auckland Cancer Society Research Centre, University of Auckland, Auckland, New Zealand; ^3^ Department of Chemistry, University of Otago, Dunedin, New Zealand

**Keywords:** anticancer activity, structural characterization, ligand exchange reactions, bioorganometallics, heterodimetallic complexes

## Abstract

Multimetallic complexes have been shown in several examples to possess greater anticancer activity than their monometallic counterparts. The increased activity has been attributed to altered modes of action. We herein report the synthesis of a series of heterodimetallic compounds based on a ditopic ligand featuring 2-pyridylimine chelating motifs and organometallic half-sandwich moieties. The complexes were characterized by a combination of ^1^H NMR spectroscopy, electrospray ionization mass spectrometry, elemental analysis and single crystal X-ray diffraction. Investigations into the stability of representative complexes in DMSO-*d*_*6*_ and 10% DMSO-*d*_*6*_/D_2_O revealed the occurrence of solvent-chlorido ligand exchange. Proliferation assays in four human cancer cell lines showed that the Os-Rh complex possessed minimal activity, while all other complexes were inactive.

## 1 Introduction

Metals play various roles in biological processes (Buccella et al., 2019), e.g., proteins often use metal centers to adopt certain structures or as catalytically active sites (Haas and Franz, 2009; Wodrich and Hu, 2017; Ghosh et al., 2021). Metal complexes, most often platinum compounds, have been used for a long time to treat cancer (Jakupec et al., 2008; Kenny and Marmion, 2019; Simpson et al., 2019; Boros et al., 2020; Tremlett et al., 2021). In addition to the clinically successful DNA-targeting cis-, carbo- and oxaliplatin (Johnstone et al., 2014), other mononuclear species studied include Ru, Os, Rh and Ir-based compounds (Sava et al., 2003; Arango et al., 2004; Hartinger et al., 2008; Geldmacher et al., 2012; Maillet et al., 2013). The non-Pt complexes have been headlined by various Ru-based examples, including the clinically explored KP1339 (Jakupec et al., 2008; Heffeter et al., 2010).

**FIGURE 1:**
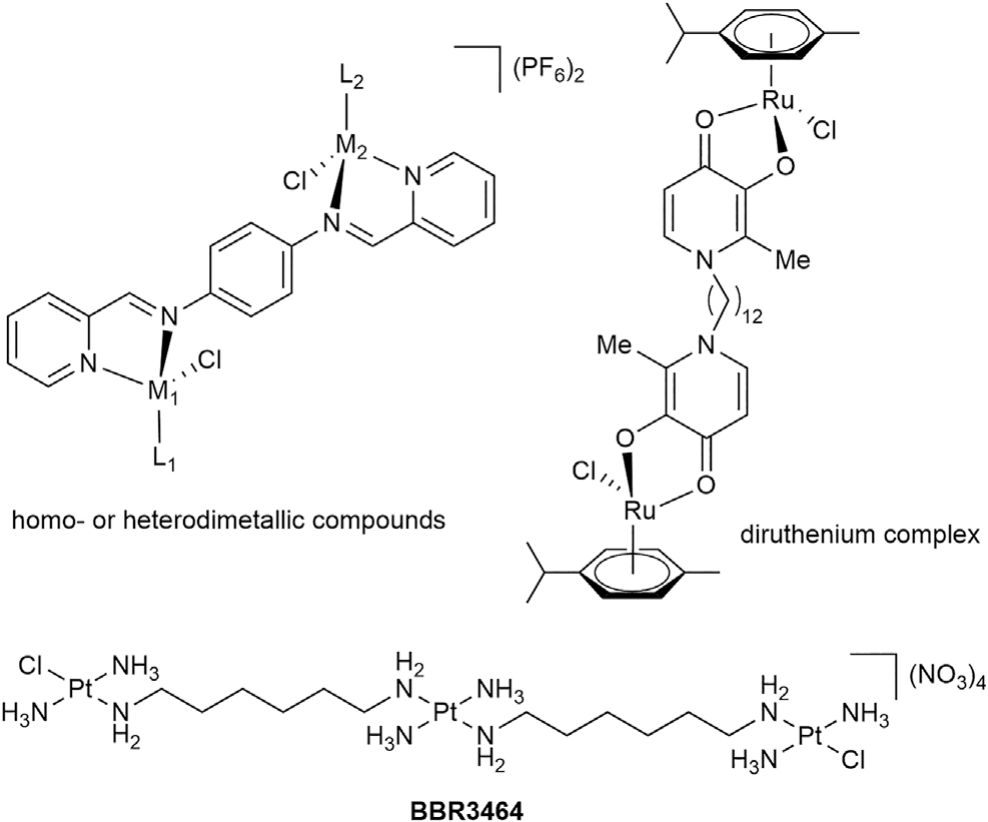
Homo- and heterometallic complexes with anticancer activity.

Synthetic attempts to link one or more metal-containing fragments have been made in order to obtain compounds with higher potency and modes of action different from those of the established anticancer agents. The tris-Pt compound BBR3464 (Figure 1) features Pt centers which contribute to DNA binding through electrostatic or covalent interactions (Manzotti et al., 2000). Indeed, the DNA binding of BBR3464 was found to be considerably different to that of cisplatin and the compound was found to show no cross-resistance in cancer cells (Manzotti et al., 2000; Ruhayel et al., 2012). Inspired by this approach, we reported organometallic compounds containing two metal centers coordinated to a maltol-derived bis(3-hydroxy-2-methyl-4-pyridone) ligand bridged by a spacer of varying length (Figure 1) (Mendoza-Ferri et al., 2008; Parveen et al., 2019b). The IC_50_ value for the dodecane-bridged bis-Ru complex in SW480 cancer cells was similar to that of platinum reference compounds and was an order of magnitude lower than of the clinically-studied Ru(III) compound KP1019 (Mendoza-Ferri et al., 2008; Mendoza-Ferri et al., 2009; Nováková et al., 2009; Nazarov et al., 2018), while the mononuclear analog gave IC_50_ values greater than 100 µM, indicating a lack of cytotoxic activity (Mendoza-Ferri et al., 2008). Multimetallic triruthenium-carbonyl clusters (Gonchar et al., 2020) have also been found to be cytotoxic, and the most active of these clusters was shown to be a system that featured a glucose-inspired phosphorus ligand. The IC_50_ values for the clusters were in the sub-µM range for wildtype and cisplatin-resistant human ovarian cancer cells (Gonchar et al., 2020). Further to this triruthenium cluster, we have investigated the use of easily synthesized ditopic ligands to form homodimetallic complexes (Figure 1, top left). These structures were important as they highlighted the possibility for the rapid formation of a range of homo- and heterodimetallic species. Although the compounds had poor cytotoxicity in comparison to other dimetallic compounds (Steel et al., 2021), synthetic alterations to the ditopic ligand being used could easily yield more potent metal-based chemotherapeutics. Alternatively, scaffold structures can be adopted to form dimetallic species of greater complexity. Relevant examples include a trimeric scaffold consisting of alkylated 1,3,5-triaza-7-phosphaadamantane moieties or other phosphine or nitrogen donor-based ligand systems (Burgoyne et al., 2016; Batchelor et al., 2019; Curado et al., 2019). Moreover, tetra- and octanuclear Ir and Rh complexes can be created using dendritic structures that have Schiff-base ligands based on poly(propyleneimine) scaffolds (Payne et al., 2013). These metallodendrimers highlight an interesting trend whereby increasing multinuclearity resulted in an analogous increase in cytotoxicity as compared to the mono-metallic derivatives (Payne et al., 2013).

The nature of the anticancer activity observed for multimetallic compounds is likely to be different from those found for the corresponding monometallic analogs due to altered molecular modes of action, as discussed above for BBR3464 and closely related compounds (Ruhayel et al., 2012). Besides DNA, bis- and tris-Pt complexes can also bind to proteoglycans through sulfate anchoring (Gorle et al., 2018; Gorle et al., 2021). The resulting metalloshielding stops enzyme degradation and has recently been shown to be biologically relevant in cells (Gorle et al., 2018; Gorle et al., 2021). This method of template protection, a strategy analogous to complex–DNA binding, heralds a new avenue in the mechanisms of action for multimetallic compounds (Gorle et al., 2021).

Recent investigations have also demonstrated that cytotoxic efficacy can be altered when heterodimetallic systems are employed or could be used to release cytotoxic payload (Herry et al., 2019; Jain, 2019; Lisboa et al., 2020; Lisboa et al., 2021). The distinct properties of each metal center in terms of inertness, redox properties and affinity to bioligands will determine the lipophilic character and biological target binding ability of heterometallic compounds (Benjamin Garbutcheon-Singh et al., 2011; Parveen et al., 2019a; Batchelor et al., 2019). Mixing and matching these characteristics within one compound carves out new chemical space in the development of chemotherapeutic agents. For example, a Fe-Ru phosphane-bridged complex in ovarian carcinoma cell lines showed increased activity over the Ru-Ru analogue (Herry et al., 2019). The difference in activity was hypothesized to be the result of the Fe center facilitating the cellular uptake of the compound (Herry et al., 2019). Heteronuclear transition metal systems have also been investigated as theranostics (Redrado et al., 2021), in which the therapeutic can be traced *via* the diagnostic component of the molecule. Most often in theranostics, a fluorophore is conjugated to the metal-based therapeutic, for example, as in the recently reported gold- and ruthenium-based complexes with therapeutic and imaging capability (Bertrand et al., 2016). Polyaryl derivates like anthracene or pyrene are common fluorophores in theranostics, as is BODIPY (Bertrand et al., 2018). The versatility of BODIPY has allowed it to be incorporated into Pt-, Au-, Ru-, Ti- and Ir-based therapeutics (Bertrand et al., 2018). The formation of dimetallic theranostic compounds is also possible, yet slightly more complex (Bertrand et al., 2016). Two recent examples have paired tris(bipyridine)ruthenium and a Ru–porphyrin with therapeutics developed around a Au center (Bertrand et al., 2016). The range of investigations that have been conducted into the creation and use of heterodimetallic systems has highlighted the myriad of potential advantages afforded by employing compounds with different metal centers.

Herein, we report the synthesis and characterization of heterodimetallic complexes based on a symmetric ditopic 2-pyridylimine ligand. The stabilities of these complexes in DMSO and aqueous solutions were investigated and the cytotoxicities of the complexes in human cancer cells were determined.

## 2 Results and Discussion

**SCHEME 1:**
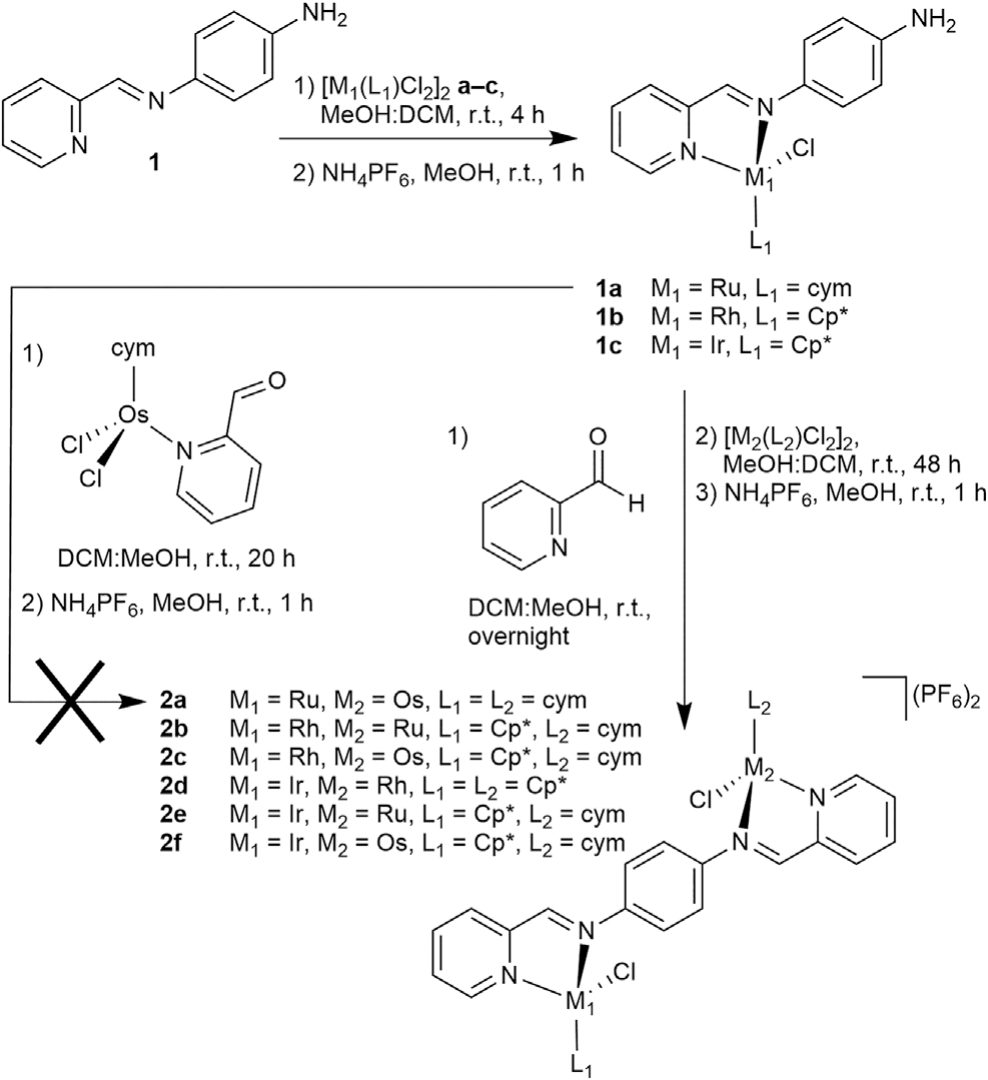
Synthetic strategy towards heterodimetallic complexes **2a**–**2f**.

Recently, we reported the preparation of homodimetallic compounds of a symmetric, ditopic 2-pyridylimine-based ligand featuring a 1,4-diaminobenzene spacer (Figure 1, L_DAB_), which is known to form stable coordination complexes (Steel et al., 2021). The formation of these di-Ru, -Os, -Rh and -Ir compounds with two different ditopic ligands highlighted the ability to reliably obtain homodimetallic compounds in good yield (Steel et al., 2021). The complexes also demonstrated a range of structural characteristics and cytotoxic activity (Steel et al., 2021). In the case of those homodimetallic complexes, the ligand was prepared before it was decorated with metal centers. This was feasible due to the symmetric nature of the target complexes. To prepare low-symmetry heterodimetallic compounds based on the same ligand the synthetic strategy had to be adapted, as the previously reported route would likely lead to a mixture of compounds. Accordingly, different pathways were explored (Scheme 1). Initially, inspired by our recent work generating heterometallic PdPt caged systems (Lisboa et al., 2020), we investigated a two-step process, in which the mononuclear precursor **1a** would be formed by reaction from mono-topic 2-pyridylimine ligand **1** with dimeric [Ru(cym)Cl_2_]_2_ **a** (cym = η^6^-*p*-cymene), followed by conversion with the preformed second metal fragment [Os(cym)(2-pyridine carboxaldehyde)Cl_2_]_2_ to target compound **2a**. Indeed, **1a** was prepared in very good yield (92%), however, the subsequent reaction with [Os(cym)(2-pyridine carboxaldehyde)Cl_2_]_2_ only provided **2a** in trace amounts (Scheme 1). In an alternative approach, **1a** was first treated with 2-pyridine carboxaldehyde to generate the second 2-pyridylimine binding site *in situ*, and the subsequent reaction with [Os(cym)Cl_2_]_2_ afforded **2a** in excellent yield (84%).

**FIGURE 2:**
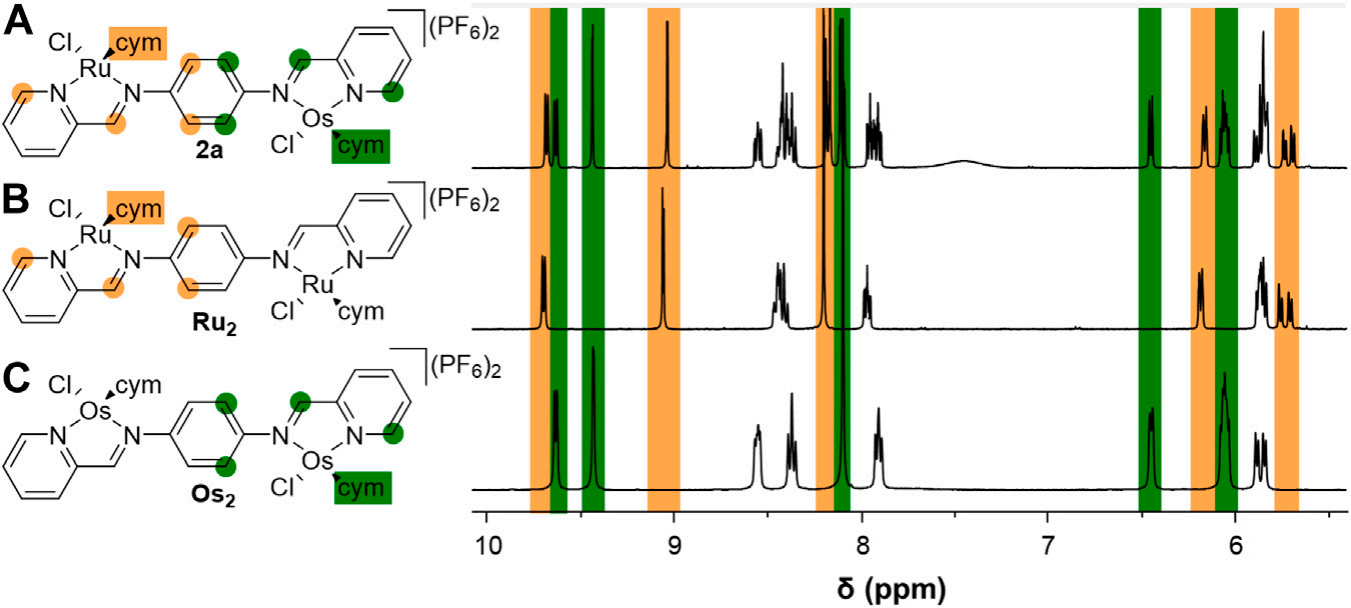
Comparison of the ^1^H NMR spectra for the heterodimetallic Ru-Os complex **2a** **(A)**, to those of the symmetric **Ru**_2_ **(B)** and **Os**_2_ **(C)** derivatives.

In the ^1^H NMR spectrum of complex **2a** peaks characteristic of π-coordinated cym were observed, with the expected integrals relative to the ligand proton signals. Notably, in contrast to the symmetric dimetallic complexes (**Ru**_2_ and **Os**_2_, Figure 2), the phenylene protons resonated as two distinct doublets due to the lower molecular symmetry, whereas the corresponding protons in the higher symmetry **Ru**_2_ and **Os**_2_ complexes were singlets (Steel et al., 2021). The incorporation of two different metals and the resulting loss of symmetry within the molecule also resulted in more complex sets of signals for the remaining protons (Figure 2 and Supplementary Figure S19). For example, the ^1^H NMR spectrum of **2a** features two sets of resonances for the α-pyridyl and imine protons of the coordinating 2-pyridylimine units (Figure 2). The chemical shift values for one set of the α-pyridyl and imine resonances match well with those observed for the homodimetallic **Ru**_2_, whilst the second set of peaks display chemical shift values that are very similar to those observed in the **Os**_2_ complexes (Figure 2). These data are consistent with the presence of two distinct 2-pyridylimine units in **2a**, one coordinated to a [Ru(cym)Cl] fragment and the second binding to an [Os(cym)Cl] unit. In the electrospray ionization (ESI) mass spectrum of **2a**, the pseudomolecular ion [M – 2PF_6_]^2+^ was observed as the base peak at *m/z* 459.0743 (*m/z*_calc_ 459.0710; Supplementary Table S1) with the isotope pattern matching that predicted for the heterodimetallic complex, in addition to [M – PF_6_]^+^ at *m/z* 1,063.1156 (*m/z*_calc_ 1,063.1067).

**FIGURE 3:**
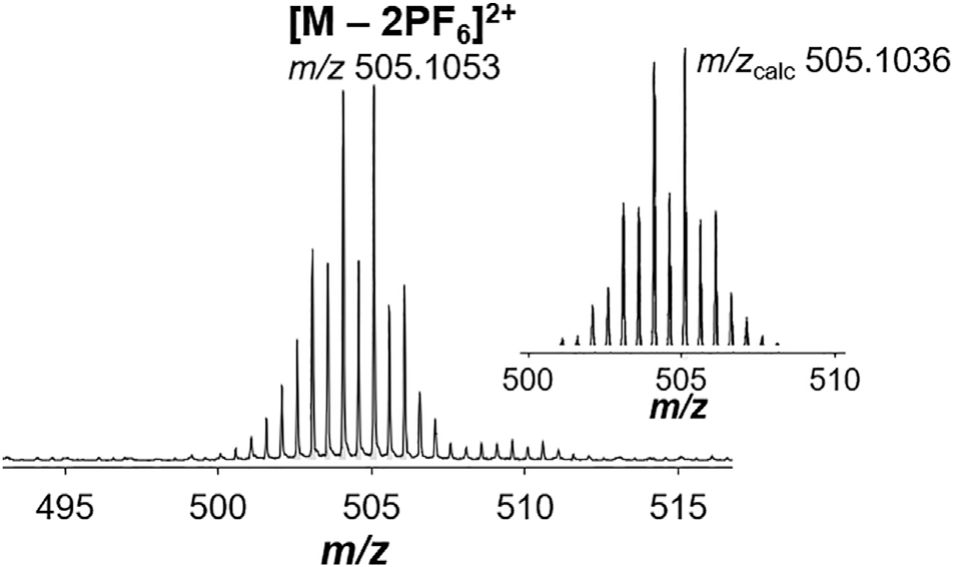
ESI-mass spectrometry data for the Os-Ir complex **2f** compared to the calculated isotope pattern for the [M – 2PF_6_]^2+^ cation.

The heterodimetallic compounds **2b**–**2f** were also synthesized in good yields (Scheme 1; 42–67%) from the mononuclear precursors **1b** (for **2b** and **2c**) and **1c** (for **2d**–**2f**) using the same strategy as for **2a** and the purity was confirmed by elemental analysis. We considered the relative labilities of the metal centers in the synthetic strategy to optimize the purity, as substitution of the metal center may occur *in situ* and result in the formation of undesired homodimetallic species. To mitigate the possibility of this occurring, the relatively inert Ir precursor **1c** was selected for use in the first reaction step when preparing **2d**–**2f**. ^1^H NMR spectra of **2b**–**2f** were all indicative of formation of the heterodimetallic complexes (Supplementary Figure S19). By comparison with the equivalent homodimetallic complexes, in particular the protons adjacent to the pyridine and imine nitrogen atoms shifted characteristically depending on the nature of the metal center. ESI-mass spectrometry (MS) was used to characterize the complexes further and the mass spectra of **2b** and **2d**–**2f** featured signals assignable to the [M – PF_6_]^+^ and [M – 2PF_6_]^2+^ pseudomolecular ions (Supplementary Table S1), with the latter usually being present in higher abundance. Each of these peaks showed clearly the isotope pattern expected for the heterodimetallic complex (compare Figure 3 for **2f**). In addition to these ions, we often detected, and in particular for **2c**, some single imine hydrolysis of the ligand, resulting in the loss of a pyridyl fragment, one of the metal centers and the respective π-bound ligand moiety, giving peaks corresponding to the mononuclear species [M – C_6_H_5_N – M(L)Cl – 2PF_6_]^+^. It should be noted that usually we detected ions that could be assigned to both metal moieties of the heterodimetallic complexes.

**FIGURE 4:**
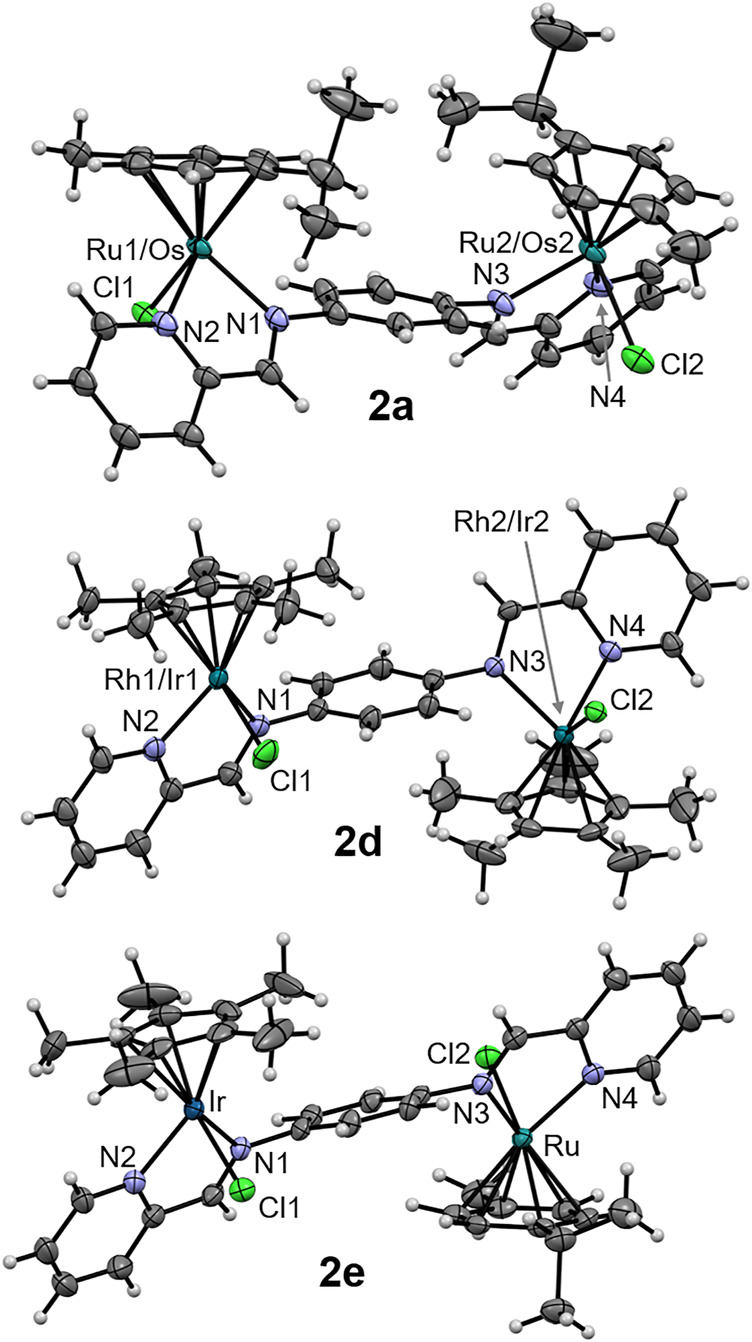
ORTEP representation of one of the two enantiomers of complexes **2a**, **2d** and **2e** drawn at 50% probability level. Any co-crystallized solvent molecules and hexafluorophosphate counterions have been omitted for clarity.

**FIGURE 5:**
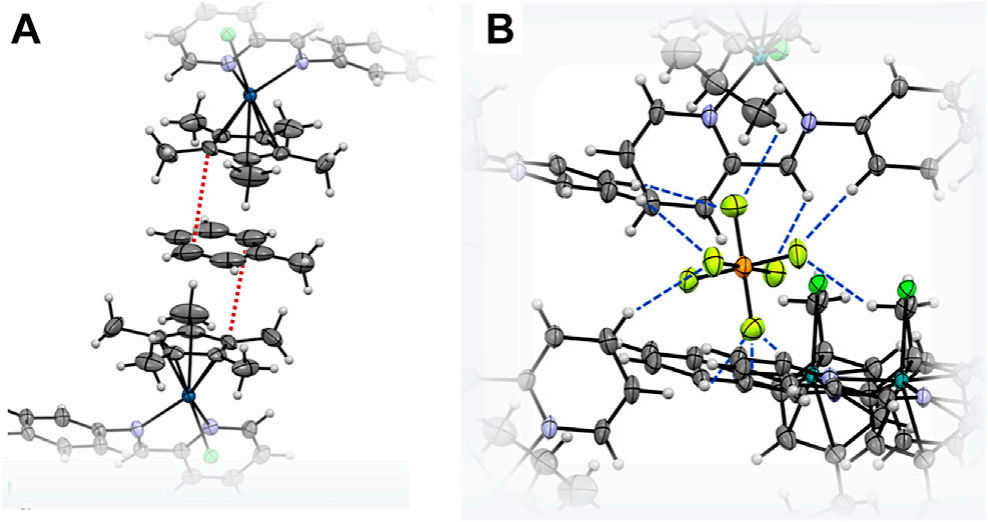
ORTEP representation of **(A)** co-crystallized toluene π-bonding with the Cp* ligands of two adjacent molecules of **2d**, and **(B)** H-bonding network around a PF_6_^−^ counteranion and **2a** molecules. The shortest C–C distances at 3.604 Å in **(A)** are highlighted by red dashed lines while the H bonds in **(B)** are indicated in blue.

The molecular structures of the complexes **2a**, **2d** and **2e** were determined by single crystal X-ray diffraction studies (Figure 4). The single crystals were grown *via* the slow diffusion of toluene into a saturated solution of the respective complex in acetonitrile. All complexes have a triclinic crystal system and crystallized in the *P*-1 space group. While **2a** co-crystallized with an acetonitrile molecule, the structure of **2d** featured a disordered toluene molecule which was found sandwiched between the Cp* ligands of two neighboring molecules of **2d** (Figure 5A). The molecular structure of **2e** also contains a strongly disordered toluene molecule, which was excluded from the final refinement (Supplementary Figure S20, Supplementary Table S2). The structure features a co-crystallized water molecule, resulting in hydrogen bonding with the chlorido ligand coordinated to the Ru center, [d(Cl2–O) = 3.205 Å], and one of the PF_6_^−^ counterions (d(F10–O) = 2.929 Å). Furthermore, in all three structures, the PF_6_^−^ counterions were extensively involved in H bonding networks with several neighboring complex molecules (Figure 5B for that of **2a**). In the cases of **2a** (with cym ligands coordinated to both Ru and Os) and **2d** (with Cp* ligands coordinated to both Rh and Ir), the metal centers were statistically distributed between the two positions in the crystal lattice; hence, it was not possible to distinguish between the two M(cym) or M(Cp*) moieties in the molecular structures. In contrast, in the structure of **2e**, the Ru and Ir centers could be unambiguously distinguished based on their different π-bound ligands, *i.e.*, cym and Cp*, respectively. For **2a** and **2d**, two independent molecules with *R*,*R* and *S*,*S* configurations at the metal centers were found in the unit cell, whereas in **2e** the two molecules had *R*,*S* and *S*,*R* configurations. For **2d** and **2e** the metal centers were arranged on opposite faces of the ligand whereas in **2a** the M(cym) moieties were found on the same face of the phenylene spacer (Table 1). In contrast to **2e**, the statistical distribution of the metal centers in **2a** and **2d** over the two sites, makes it impossible to determine accurately the M–donor atom bond lengths (Table 1).

**TABLE 1:** Key bond lengths (Å) in the molecular structures of **2a**, **2d** and **2e**.

Bonds/bond lengths (Å)	2a		2d		2e	
	M1	M2	M1	M2	Ru	Ir
M–Cl	2.4008 (11)	2.3852 (13)	2.4052 (9)	2.4138 (8)	2.3738 (8)	2.3938 (8)
M–N	2.087 (4)	2.104 (4)	2.098 (3)	2.116 (3)	2.080 (3)	2.083 (3)
M–N	2.090 (4)	2.100 (4)	2.097 (3)	2.101 (3)	2.088 (3)	2.086 (3)

**FIGURE 6:**
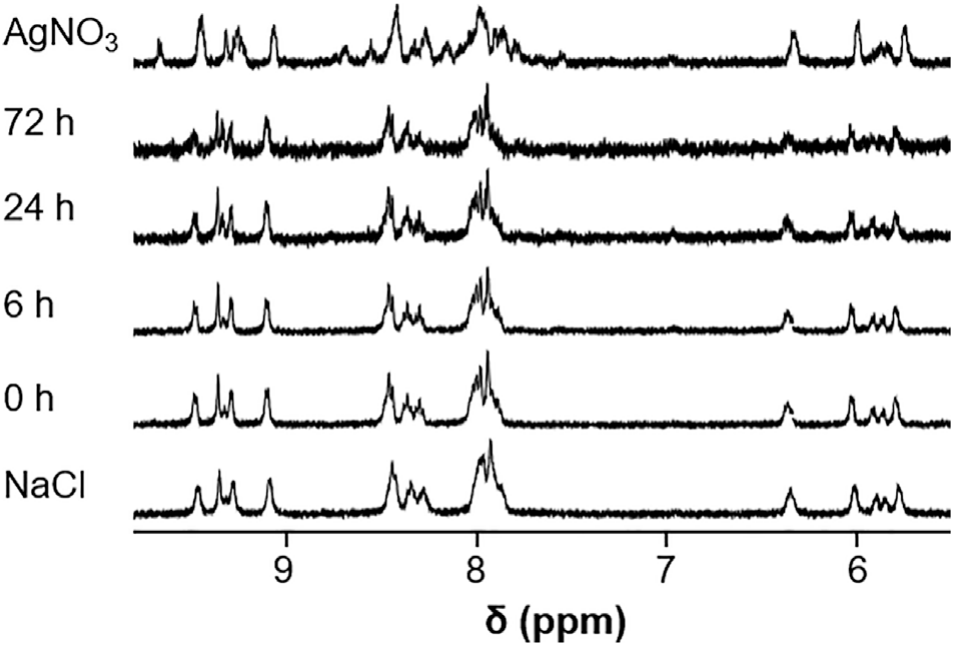
^1^H NMR spectra of complex **2f** recorded in 10% DMSO-*d*_*6*_/D_2_O over a period of 72 h as well as after addition of AgNO_3_ (2 equiv.) or NaCl (100 mM).

Imines can hydrolyze in aqueous solution into the respective amine and aldehyde building blocks, and metal complexes may undergo ligand exchange reactions, especially in the presence of coordinating solvents like DMSO which is often used for the preparation of stock solutions for biological assays. Therefore, the stabilities of the representative complexes Os-Rh **2c** and Os-Ir **2f** were analyzed by ^1^H NMR spectroscopy over a 72 h time period in DMSO-*d*_*6*_ and 10% DMSO-*d*_*6*_/D_2_O. When **2c** was dissolved in DMSO-*d*_*6*_, a second set of peaks appeared over a 72 h period suggesting coordination of DMSO to the Os center (Supplementary Figure S21). In the case of the Os-Ir compound **2f**, minor changes were observed, but these were not as pronounced as those observed for **2c** (Supplementary Figure S22). Interestingly, for the Cp* signals in the spectra of both compounds, we observed changes in the aliphatic region which require further investigations (Banerjee et al., 2018; Lee B. Y. T. et al., 2021; Lee B. et al., 2021). In 10% DMSO-*d*_*6*_/D_2_O, the spectra of **2f** changed over time which was suppressed by the addition of 100 mM NaCl, to approximate the standard chloride concentration in blood. In contrast, treatment with AgNO_3_ resulted in precipitation of AgCl and significant alteration of the spectra, most likely owing to the formation of the aqua complex after abstraction of the chlorido ligand (Figure 6). An analogous pattern was observed in the case of **2c**, with the addition of AgNO_3_ causing a precipitate of AgCl and a noticeable change in the shifts of the aromatic protons (Supplementary Figure S23). Note that in both experiments, precipitation occurred over the 72 h time period which resulted in spectra with lower signal-to-noise ratios which made the interpretation of the spectra more difficult.

All complexes were investigated for their cytotoxic activity in HCT116 (human colon cancer cell line), SW480 (human colon adenocarcinoma cell line), NCI-H460 (human non-small-cell lung cancer cell line) and SiHa (human cervical cancer cell line) cancer cell lines with the sulforhodamine B assay. Compounds that gave mean growth inhibitory concentrations (IC_50_) > 100 µM were deemed to be inactive in the respective cell line. The IC_50_ values obtained for each complex have been summarized in Table 2 and compared to the bidentate pyridyl-imine-based ligand **2** and the analogous homodimetallic complexes in the same cell lines and under the same conditions (Steel et al., 2021). Ligand **2** demonstrated only very moderate activity in HCT116, NCI-H460 and SiHa cells, while it was inactive in SW480. The formation of coordination compounds of **2** unfortunately did not significantly alter the overall picture, and only for Os-Rh compound **2c** IC_50_ values could be determined, which only showed moderate cytotoxicity. This reflects to a large extent the observations we made for the analogous homodiatomic complexes **Ru**_2_, **Os**_2_, **Rh**_2_ and **Ir**_2_ (Steel et al., 2021). While we and others have reported significant improvements in terms of cytotoxic effects when two or more metal centers are linked in a single molecule (Mendoza-Ferri et al., 2009), it is apparent that the incorporation of multiple metal centers in a single molecule does not necessarily result in high antiproliferative activity. The biological effect is significantly influenced by the ligand system used. This may in part be because of the structure of the ligand but may also be related to the stability of the formed complexes, with possibly even the ligands being the cytotoxic species upon cleavage of the metal centers from the coordinating motif. In any case, cellular accumulation of the active species is a prerequisite to observe biological activity which may be the reason for the low activity of this compound type (Jakupec et al., 2005). However, the low antiproliferative activity of the investigated complexes does not warrant further biological investigations.

**TABLE 2:** IC_50_ values (µM) complexes **2a**–**2f** in HCT116, NCI-H460, SiHa and SW480 cancer cell lines expressed as mean ± standard error (*n* = 3), in comparison to ligand **2** and the homodimetallic analogs **Ru**_2_, **Os**_2_, **Rh**_2_ and **Ir**_2_ (Steel et al., 2021).

Compound	IC_50_ value/μM			
	HCT116	NCI-H460	SiHa	SW480
**2**	55 ± 20	57 ± 6	88 ± 4	>100
**Ru**_2_	>100	>100	>100	>100
**Os**_2_	>100	>100	>100	>100
**Rh**_2_	70 ± 29	61 ± 13	70 ± 1	73 ± 4
**Ir**_2_	>100	>100	>100	>100
**2a**	>100	>100	>100	>100
**2b**	>100	>100	>100	>100
**2c**	>100	56 ± 12	77 ± 10	45 ± 9
**2d**	>100	>100	>100	>100
**2e**	>100	>100	>100	>100
**2f**	>100	>100	>100	>100

## 3 Conclusion

In an attempt to improve the anticancer activity of organometallic anticancer agents, we designed and prepared heterodimetallic compounds based on a ditopic symmetric ligand. The synthetic strategy was developed by taking into consideration the labilities of the intermediate complexes to facilitate the isolation of the target heterodimetallic compounds in pure form. ^1^H NMR spectroscopy in acetone-*d*_*6*_ enabled the ligand components coordinated to the respective metal center to be distinguished, while ESI-MS confirmed the formation of the target compounds. Single crystal X-ray diffraction analysis showed statistical distribution of the metal centers in the heterometallic Ru/Os(cym) and Rh/Ir(Cp*) compounds **2a** and **2d**, while in case of complex **2e** the Ru(cym) and Ir(Cp*) moieties were easily distinguishable based on the π-bound ligand. Compounds **2c** and **2f** were investigated for stability in solution and both were found to undergo ligand exchange reactions, although at different rates. In assays to investigate antiproliferative activity, only **2c** showed very moderate potency. While the antiproliferative activity of the current complexes was only modest, the robust, facile and modular nature of the method to form these heterodimetallic compounds means that a wide range of systems could be synthesized and examined for biological activity. Furthermore, this method to heterodimetallic complexes may also find applications in the development of new catalysts (Mata et al., 2014; Gaston et al., 2021; Patra and Maity, 2021).

## 4 Experimental Section

### 4.1 Materials and Methods

All air and moisture-sensitive reactions were carried out under a nitrogen (N_2_) atmosphere, and light sensitive reactions were protected from photolytic degradation by covering the apparatus in aluminum foil. Chemicals and solvents purchased from commercial suppliers were used without further purification. Solvents were dried prior to use when necessary. Solvents were evaporated under reduced pressure using a rotary evaporator. The precursor complexes [Ru(cym)Cl_2_]_2_ **a** (Bennett and Smith, 1974), [Rh(Cp^*^)Cl_2_]_2_ **b** (Vogt et al., 2005; Nejman et al., 2015), [Ir(Cp^*^)Cl_2_]_2_ **c** (Vogt et al., 2005; Jakoobi et al., 2017) and [Os(cym)Cl_2_]_2_ **d** (Peacock et al., 2007) were prepared according to literature procedures.

1D [^1^H, and ^13^C{^1^H} DEPT-Q, ^31^P{^1^H}] and 2D (^1^H-^1^H COSY, ^1^H-^1^H NOESY, ^1^H-^13^C HSQC, ^1^H-^13^C HMBC) NMR spectra were recorded on Bruker DRX 400 MHz NMR spectrometers at 25 °C. The measurement frequencies for ^1^H, ^13^C{^1^H}, and ^31^P{^1^H} NMR spectra were 399.89, 100.55 and 161.85 MHz, respectively. Deuterated chloroform, acetone-*d*_*6*_, D_2_O, and DMSO-*d*_*6*_ were used as NMR solvents and the chemical shifts are reported relative to the residual solvent peaks. The mass spectra were recorded on a Bruker micrOTOF-Q II ESI-MS in positive ion mode. X-ray diffraction measurements of single crystals were conducted on a Rigaku Oxford Diffraction XtaLABSynergy-S single-crystal diffractometer with a PILATUS 200 K hybrid pixel array detector using Cu Kα radiation (λ = 1.54184 Å). The structure solution and refinements were performed with the SHELXS-97, SHELXL-2016 (Sheldrick, 2008) and Olex2 program packages (Dolomanov et al., 2009; Bourhis et al., 2015). Molecular structures were visualized using Mercury 4.0.0. Elemental analyses were carried out on the vario EL cube CHNOS Elemental analyzer at the University of Auckland for Ru, Rh, and Ir complexes, and at the Campbell Microanalytical Laboratory, the University of Otago for Os complexes.

### 4.2 Syntheses

#### 4.2.1 General Procedure for the Synthesis of Mononuclear Complexes **1a**, **1c** and **1d**

**FIGURE 7:**
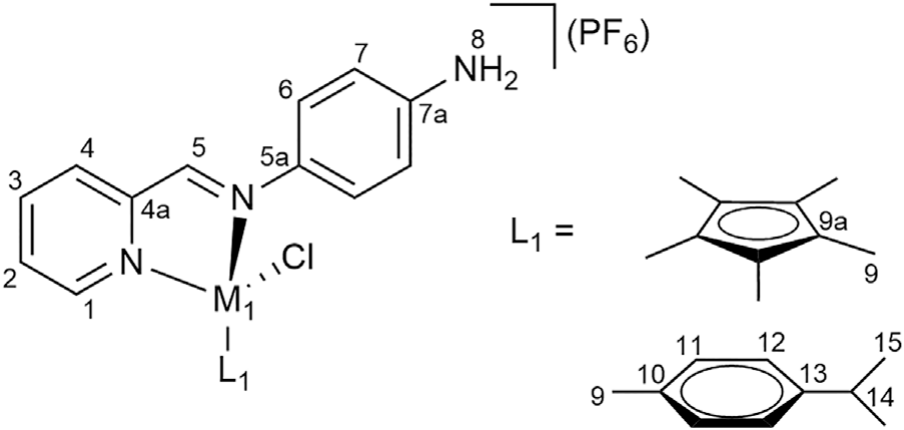
NMR numbering scheme for complexes **1a**–**1c**.

Pyridyl-imine ligand **1** (1.0 eq) was added to a solution of dimeric precursor **a**, **c** or **d** (0.5 eq) in DCM:MeOH (15 ml, 1:1) and stirred at r. t. for 4 h. The solvent was then removed under reduced pressure. A solution of ammonium hexafluorophosphate (NH_4_PF_6_, 20 eq) in MeOH (20 ml) was added to a solution of the crude product in MeOH (20 ml) and the resulting mixture was stirred for 1 h. The solution was concentrated under reduced pressure and diethyl ether was added. The resulting precipitate was collected by filtration, washed with cold MeOH and dried. The crude product was dissolved in DCM (80 ml), filtered and the solvent was removed from the filtrate under reduced pressure to yield the products (Figure 7 for the NMR numbering scheme) after drying the residue *in vacuo*.

[Chlorido(η^6^-p-cymene)(4-((pyridine-2-ylmethylene)amino)aniline)ruthenium(II)] hexafluorophosphate **1a**.

**1** (161 mg, 0.10 mmol), [Ru(cym)Cl_2_]_2_ **a** (250 mg, 0.05 mmol) and NH_4_PF_6_ (1.33 g, 1.02 mmol) to afford **1a** as a dark red powder (461 mg, 92%). m. p.: 104.8–118.2°C (decomposition). ^1^H NMR (acetone-*d*_*6*_): δ 9.55 (d, ^3^ *J* = 2 Hz, 1H, H-1), 8.73 (s, 1H, H-5), 8.32–8.24 (m, 2H, H-2,3), 7.84–7.79 (m, 1H, H-4), 7.69–7.64 (m, 2H, H-6), 6.87–6.81 (m, 2H, H-7), 6.11 (d, ^3^ *J* = 3 Hz, 1H, H-11), 5.77 (d, ^3^ *J* = 3 Hz, 1H, H-12), 5.72 (d, ^3^ *J* = 3 Hz, 1H, H-11), 5.66 (d, ^3^ *J* = 3 Hz, 1H, H-12), 5.45 (s, 2H, H-8), 2.68 (sept, ^3^ *J* = 7 Hz, 1H, H-10), 2.30 (s, 3H, H-13), 1.15–1.07 ppm (m, 6H, H-9). ^13^C{^1^H} DEPT-Q (acetone-*d*_*6*_): δ 162.6 (C-5), 156.6 (C-1), 156.4 (C-4a), 151.9 (C-7a), 142.6 (C-5a), 140.6 (C-2,3), 129.6 (C-2,3), 128.9 (C-4), 125.2 (C-6), 114.6 (C-7), 106.8 (C-13), 104.8 (C-10), 87.9 (C-12), 87.3 (C-12), 86.7 (C-11), 85.9 (C-11), 31.9 (C-14), 22.2 (C-15), 18.7 ppm (C-9). ^31^P{^1^H} NMR (acetone-*d*_*6*_): δ –144.29 ppm (sept, ^*2*^ *J* = 711 Hz, PF_6_^−^). MS (ESI^+^): *m/z* 468.0788 [M – PF_6_]^+^ (m_calc_ = 468.0775).

[Chlorido(η^5^-pentamethylcyclopentadienyl)(4-((pyridine-2-ylmethylene)amino)aniline)rhodium(III)] hexafluorophosphate **1b.**

The synthesis was performed according to the general procedure using **1** (128 mg, 0.65 mmol), [Rh(Cp*)Cl_2_]_2_ **b** (201 mg, 0.32 mmol), and NH_4_PF_6_ (1.1 g, 6.5 mmol) to afford **1b** as a bright orange powder (335 mg, 84%). m. p.: 147 °C (clear point). ^1^H NMR (DMSO-*d*_6_): δ 8.98 (d, ^3^ *J* = 6 Hz, 1H, H-1), 8.76 (d, ^3^ *J* = 2 Hz, 1H, H-5), 8.31 (td, ^3^ *J* = 8 Hz, ^4^ *J* = 2 Hz, 1H, H-3), 8.20 (d, ^3^ *J* = 8 Hz, 1H, H-4), 7.91–7.86 (m, 1H, H-2), 7.49–7.43 (m, 2H, H-6), 6.72–6.70 (m, 2H, H-7), 5.79 (s, 2H, H-8), 1.45 ppm (s, 15H, H9). ^13^C{^1^H} DEPT-Q (DMSO-*d*_6_): δ 162.3 (C-5), 154.4 (C-4a), 152.6 (C-1), 150.6 (C-5a), 140.3 (C-3), 137.2 (C-7a), 128.9 (C-2), 128.6 (C-4), 124.1 (C-6), 113.2 (C-7), 97.0 (C-9a), 8.3 ppm (C-9). ^31^P{^1^H} NMR (DMSO-*d*_6_): δ –144.7 ppm (sept, ^*2*^ *J* = 711 Hz, PF_6_^−^). MS (ESI^+^): *m/z* 470.0899 [M – PF_6_]^+^ (m_calc_ = 470.0870).

[Chlorido(η^5^-pentamethylcyclopentadienyl)(4-((pyridine-2-ylmethylene)amino)aniline)iridium(III)] hexafluorophosphate **1c.**

The synthesis was performed according to the general procedure using **1** (282 mg, 1.40 mmol), [Ir(Cp*)Cl_2_]_2_ **c** (569 mg, 0.71 mmol), and NH_4_PF_6_ (3.5 g, 29 mmol) to afford **1c** as a dark orange powder (652 mg, 82%). m. p.: 176°C (clear point). ^1^H NMR (DMSO-*d*_6_): δ 9.14 (s, 1H, H-5), 8.97 (d, ^3^ *J* = 6 Hz, 1H, H-1), 8.33–8.25 (m, 2H, H-3/4), 7.89–7.83 (m, 1H, H-2), 7.41 (d, ^3^ *J* = 9 Hz, 2H, H-6), 6.68 (d, ^3^ *J* = 9 Hz, 2H, H-7), 5.80 (s, 2H, H-8), 1.44 ppm (s, 15H, H9). ^13^C{^1^H} DEPT-Q (DMSO-*d*_6_): δ 163.8 (C-5), 156.0 (C-4a), 152.1 (C-1), 150.8 (C-5a), 140.5 (C-3/4), 137.6 (C-7a), 129.5 (C-2), 128.7 (C-3/4), 124.2 (C-6), 113.1 (C-7), 89.6 (C-9a), 8.0 ppm (C-9). ^31^P{^1^H} NMR (DMSO-*d*_6_): δ –144.7 ppm (sept, ^*2*^ *J* = 711 Hz, PF_6_^−^). MS (ESI^+^): *m/z* 560.1440 [M – PF_6_]^+^ (m_calc_ = 560.1444).

#### 4.2.2 General Procedure for the Syntheses of Heterodimetallic Complexes **2a**–**2f**

**FIGURE 8:**
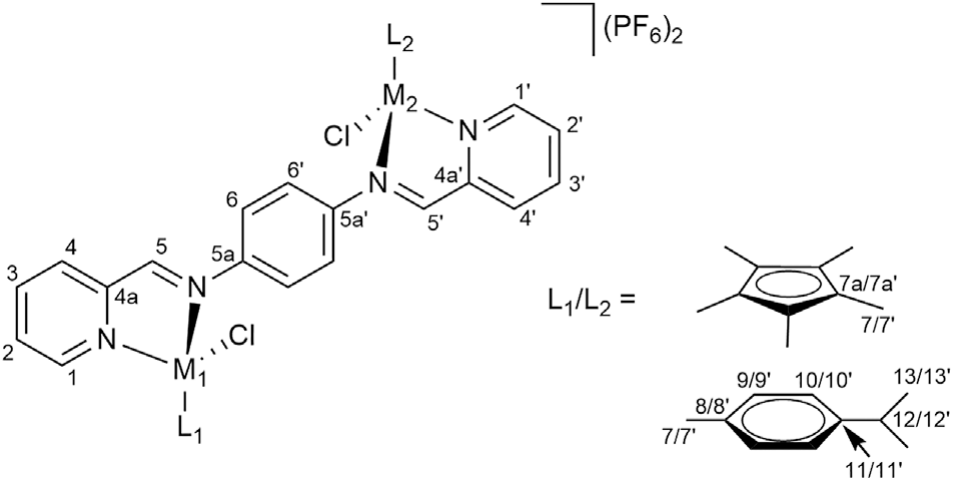
NMR numbering scheme for complexes **2a**–**2f**.

2-Pyridine carboxaldehyde (1 eq) was added to a solution of precursor complexes **1a**–**1c** (1 eq) in DCM:MeOH (30 ml, 1:1) and stirred at r. t. overnight. Dimeric precursor **a**, **b** or **d** (0.5 eq) was added and the resulting mixture was stirred for *ca*. 48 h. The solvent was removed under reduced pressure. A solution of NH_4_PF_6_ (20 eq) in MeOH (20 ml) was added to the solution of the crude product in MeOH (20 ml) and the resulting mixture was stirred for 1 h. The solution was concentrated under reduced pressure and diethyl ether was added. The precipitate was filtered, washed with cold MeOH and dried. The crude product was dissolved in acetone (40 ml), filtered and the solvent was removed from the filtrate under reduced pressure, yielding pure complexes (Figure 8 for the NMR numbering scheme) after drying the residue *in vacuo*.

[Chlorido(η^6^-p-cymene)osmium(II)](N,N'-(1,4-phenylene)(bis(1-(pyridin-2-yl)(methanimine)-*κ* ^2^N,N')[chlorido(η^6^-p-cymene)ruthenium(II)] hexafluorophosphate **2a**.

The synthesis was performed according to the general procedure using **1a** (240 mg, 0.4 mmol), 2-pyridine carboxaldehyde (37 µL, 0.39 mmol), [Os(cym)Cl_2_]_2_ (158 mg, 0.20 mmol) and NH_4_PF_6_ (1.30 g, 8.0 mmol) to afford **2a** as a dark red-brown powder (397 mg, 84%). m. p.: 161.2–173.5°C (decomp.). ^1^H NMR (acetone-*d*_*6*_): δ 9.71 (d, ^3^ *J* = 2 Hz, 1H, H-1), 9.66 (d, ^3^ *J* = 3 Hz, 1H, H-1'), 9.46 (s, 1H, H-5'), 9.06 (s, 1H, H-5), 8.60–8.51 (m, 1H, H-3'), 8.48–8.36 (m, 3H, H-3,4', 4), 8.23–8.18 (m, 2H, H-6), 8.15–8.10 (m, 2H, H-6'), 8.00–7.91 (m, 2H, H-2,2'), 6.47 (d, ^3^ *J* = 4 Hz, 1H, H-9'), 6.19 (d, ^3^ *J* = 4 Hz, 1H, H-9), 6.10–6.02 (m, 2H, H-9'/10'), 5.91–5.83 (m, 3H, H-9, 10', 10), 5.71 (dd, ^3^ *J* = 8 Hz, ^4^ *J* = 3 Hz, 1H, H-10), 2.78 (m, 1H, H-12), 2.64 (sept, ^3^ *J* = 7 Hz 1H, H-12'), 2.40 (d, ^3^ *J* = 3 Hz, 3H, H-7), 2.33 (d, ^3^ *J* = 3 Hz, 3H, H-7'), 1.18–1.12 (m, 6H, H-13), 1.11–1.06 ppm (m, 6H, H-13'). ^13^C{^1^H} DEPT-Q (acetone-*d*_*6*_): δ 170.0 (C-5'), 169.1 (C-5), 157.1 (C-1), 156.7 (C-1'), 155.8 (C-4a'), 154.2 (C-5a'), 154.0 (C-5a), 153.9 (C-4a), 141.2 (C-4,4'), 141.0 (C-4,4'), 131.5 (C-3), 131.3 (C-3'), 131.1 (C-2'), 130.2 (C-2), 125.4 (C-6'), 124.9 (C-6), 107.9 (C-11), 104.9 (C-8), 99.6 (C-11'), 98.8 (C-8'), 87.8 (C-9), 86.8 (C-9,10), 86.4 (C-10), 79.7 (C-9'), 79.1 (C-9'/10'), 77.3 (C-9'/10'), 76.9 (C-10'), 32.2 (C-12), 32.0 (C-12'), 22.6 (C-13'), 22.2 (C-13), 18.9 ppm (C-7,7'). ^31^P{^1^H} NMR (acetone-*d*_*6*_): δ –144.37 ppm (sept, ^*2*^ *J* = 707 Hz, PF_6_^−^). MS (ESI^+^): *m/z* 459.0721 [M – 2PF_6_]^2+^ (m_calc_ = 459.0723). EA calculated for C_38_H_42_Cl_2_F_12_N_4_P_2_RuOs**·**0.67NH_4_PF_6_: C 34.69%, H 3.42%, N 4.97%. Found: C 35.03%, H 3.50%, N 5.07%.

[Chlorido(η^5^-pentamethylcyclopentadienyl)rhodium(III)](N,N'-(1,4-phenylene)(bis(1-(pyridin-2-yl)(methanimine)-*κ* ^2^N,N')[chlorido(η^6^-p-cymene)ruthenium(II)] hexafluorophosphate **2b**.

The synthesis was performed according to the general procedure using **1b** (165 mg, 0.27 mmol), 2-pyridine carboxaldehyde (26 µL, 0.27 mmol), [Ru(cym)Cl_2_]_2_ (82 mg, 0.14 mmol) and NH_4_PF_6_ (872 mg, 5.35 mmol) to afford **2b** as a yellow powder (152 mg, 51%). m. p.: 141°C (clear point). ^1^H NMR (acetone-*d*_*6*_): δ 9.69 (d, ^3^ *J* = 6 Hz, 1H, H-1'), 9.27–9.23 (m, 1H, H-1), 9.10–9.04 (m, 2H, H-5/5'), 8.49–8.37 (m, 4H, H-3/3'/4/4'), 8.25–8.18 (m, 1H, H-6'), 8.14–8.05 (m, 3H, H-2/6), 7.98–7.93 (m, 1H, H-2'), 6.21–6.16 (m, 1H, H-9'), 5.89–5.69 (m, 3H, H-8'/9'), 5.70 (d, ^3^ *J* = 6 Hz, 1H, H-8'), 2.74 (m, 1H, H-10'), 2.34–2.30 (m, 3H, H-7'), 1.69–1.63 (m, 15H, H-7), 1.20–1.12 ppm (m, 6H, H-11'). ^13^C{^1^H} DEPT-Q (acetone-*d*_*6*_): δ 169.1 (C-5/5'), 157.0 (C-1'), 153.9 (C-1), 153.7 (C-4a/4a'/5a), 150.7 (C-4a/4a'), 150.6 (C-5a'), 141.6 (C-3/3'), 141.0 (C-3/3'), 131.5 (C-4/4'), 131.4 (C-4/4'), 131.2 (C-2), 130.2 (C-2'), 125.1 (C-6), 125.0 (C-6'), 107.8 (C-11'), 105.0 (C-8'), 98.7 (C-7a), 87.7–86.2 (C-9'/10'), 32.0 (C-12'), 22.3 (C-12'), 19.0 (C-7'), 9.0 ppm (C-7). ^31^P{^1^H} NMR (acetone-*d*_*6*_): δ –144.3 ppm (sept, ^*2*^ *J* = 708 Hz, PF_6_^−^). MS (ESI^+^): *m/z* 470.0870 [M – 2PF_6_ – C_6_NH_5_ – RuCl(*p*-cym)]^+^ (m_calc_ = 470.0870). EA calculated for C_38_H_43_Cl_2_F_12_RhN_4_P_2_Ru**·**0.2NH_4_PF_6_: C 39.57%, H 3.84%, N 5.10%. Found: C 39.67%, H 4.20%, N 4.80%.

[Chlorido(η^6^-p-cymene)osmium(II)](N,N'-(1,4-phenylene)(bis(1-(pyridin-2-yl)(methanimine)-*κ* ^2^N,N')[chlorido(η^5^-pentamethylcyclopentadienyl)rhodium(III)] hexafluorophosphate **2c.**

The synthesis was performed according to the general procedure using **1b** (153 mg, 0.25 mmol), 2-pyridine carboxaldehyde (24 µL, 0.25 mmol), [Os(cym)Cl_2_]_2_ (98 mg, 0.13 mmol) and NH_4_PF_6_ (808 mg, 4.96 mmol) to afford **2c** as an orange powder (127 mg, 42%). m. p.: 154°C (clear point). ^1^H NMR (acetone-*d*_*6*_): δ 9.64 (d, ^3^ *J* = 6 Hz*,* 1H, H-1'), 9.50 (s, 1H, H-5'), 9.26 (d, ^3^ *J* = 6 Hz, 1H, H-1), 9.06 (m, 1H, 1H, H-5), 8.55 (m, 1H, H-4'), 8.47–8.43 (m, 2H, H-3/4), 8.37 (m, 1H, H-3'), 8.16–8.07 (m, 5H, H-2/6/6'), 7.93–7.89 (m, 1H, H-2'), 6.49–6.45 (m, 1H, H-8'), 6.09–5.98 (m, 2H, H-8'/9'), 5.92–5.84 (m, 1H, H-9'), 2.64 (sept, ^3^ *J* = 7 Hz, 1H, H-10'), 2.41–2.37 (m, 3H, H-7'), 1.68–1.64 (m, 15H, H-7), 1.27–1.06 ppm (m, 6H, H-11'). ^13^C{^1^H} DEPT-Q (acetone-*d*_*6*_): δ 170.0 (C-5'), 169.2 (C-5), 157.2 (C-4a'), 156.7 (C-1'), 153.9 (C-1), 153.7 (C-5a/5a'), 150.9 (C-5a/5a'), 141.6 (C-3), 141.1 (C-3'), 131.4 (C-4), 131.3 (C-4') 131.2 (C-2), 131.1 (C-2'), 125.5 (C-6/6'), 124.9 (C-6/6'), 99.5 (C-11'), 99.3 (C-8'), 98.6 (C-7a), 79.6–76.7 (C-9'/10'), 32.2 (C-12'), 22.6 (C-13'), 18.9 (C-7'), 9.0 ppm (C-7). ^31^P{^1^H} NMR (acetone-*d*_*6*_): δ –144.3 ppm (sept, ^*2*^ *J* = 708 Hz, PF_6_^−^). MS (ESI^+^): *m/z* 470.0817 [M – 2PF_6_ – C_6_NH_5_ – OsCl(*p*-cym)]^+^ (m_calc_ = 470.0870). EA calculated for C_38_H_43_Cl_2_F_12_RhN_4_P_2_Os: C 37.73%, H 3.58%, N 4.63%. Found: C 37.77%, H 3.39%, N 4.44%.

[Chlorido(η^5^-pentamethylcyclopentadienyl)iridium(III)](N,N'-(1,4-phenylene)(bis(1-(pyridin-2-yl)(methanimine)- *κ* ^2^N,N')[chlorido(η^5^-pentamethylcyclopentadienyl)rhodium(III)] hexafluorophosphate **2d.**

The synthesis was performed according to the general procedure using **1c** (174 mg, 0.25 mmol), 2-pyridine carboxaldehyde (24 µL, 0.25 mmol), [Rh(Cp^*^)Cl_2_]_2_ (76 mg, 0.13 mmol) and NH_4_PF_6_ (806 mg, 5.0 mmol) to afford **2d** as a dark yellow powder (170 mg, 58%). Single crystals suitable for X-ray diffraction analysis were grown by slow diffusion of toluene into a saturated solution of the complex in acetonitrile. m. p.: 135 °C (clear point). ^1^H NMR (acetone-*d*_*6*_): δ 9.53–9.50 (m, 1H, H-5), 9.27–9.23 (m, 2H, H-1/1'), 9.10 (dd, ^3^ *J* = 6 Hz, ^4^ *J* = 3 Hz, 1H, H-5'), 8.59–8.55 (m, 1H, H-4), 8.49–8.41 (m, 3H, H-3/3'/4'), 8.16–8.05 (m, 6H, H-2/2'/6/6'), 1.66–1.65 (m, 15H, H-7') 1.64–1.63 ppm (m, 15H, H-7). ^13^C{^1^H} DEPT-Q (acetone-*d*_*6*_): δ 170.6 (C-5), 169.3 (C-5'), 153.8 (C-1/1'), 153.4 (C-1/1'), 150.7 (C-4a), 150.6 (C-4a'), 141.7 (C-4/4'), 141.5 (C-3/3'), 131.8 (C-2/2'), 131.4 (C-2/2'), 131.3 (C-4') 131.2 (C-4), 125.2 (C-6/6'), 124.9 (C-6/6'), 98.7 (C-7a'), 91.4 (C-7a), 9.0 (C-7'), 8.8 ppm (C-7). ^31^P{^1^H} NMR (acetone-*d*_*6*_): δ –144.3 ppm (sept, ^*2*^ *J* = 707 Hz, PF_6_^−^). MS (ESI^+^): *m/z* 461.0794 [M – 2PF_6_]^2+^ (m_calc_ = 461.0814). 461.0794. EA calculated for C_38_H_44_Cl_2_F_12_IrN_4_P_2_Rh·0.7H_2_O: C 37.24%, H 3.74%, N 4.57%. Found: C 36.84%, H 4.11%, N 4.39%.

[Chlorido(η^5^-pentamethylcyclopentadienyl)iridium(III)](N,N'-(1,4-phenylene)(bis(1-(pyridin-2-yl)(methanimine)-*κ* ^2^N,N')[chlorido(η^6^-p-cymene)ruthenium(II)] hexafluorophosphate **2e**.

The synthesis was performed according to the general procedure using **1c** (175 mg, 0.25 mmol), 2-pyridine carboxaldehyde (24 µL, 0.25 mmol), [Ru(cym)Cl_2_]_2_ (76 mg, 0.13 mmol) and NH_4_PF_6_ (808 mg, 5.0 mmol) to afford **2e** as a dark brown powder (151 mg, 50%). Single crystals suitable for X-ray diffraction analysis were grown by slow diffusion of toluene into a saturated solution of the complex in acetonitrile. m. p.: 169°C (clear point). ^1^H NMR (acetone-*d*_*6*_): δ 9.68 (d, ^3^ *J* = 6 Hz, 1H, H-1'), 9.48 (s, 1H, H-5), 9.25 (d, ^3^ *J* = 6 Hz, 1H, H-1), 9.08 (s, 1H, H-5'), 8.54–8.45 (m, 1H, H-4), 8.46–8.37 (m, 3H, H-3/3'/4'), 8.25–8.18 (m, 2H, H-6') 8.10–8.04 (m, 3H, H-2/6), 7.97–7.92 (m, 1H, H-2') 6.21–6.15 (m, 1H, H-9'), 5.87–5.73 (m, 2H, H-8'/9'), 5.69 (d ^3^ *J* = 6 Hz, 1H, H-8'), 2.75 (m, 1H, H-10'), 2.32 (s, 3H, H-7'), 1.65–1.62 (m, 15H, H-7), 1.19–1.13 ppm (m, 6H, H-11'). ^13^C{^1^H} DEPT-Q (acetone-*d*_*6*_): δ 170.5 (C-5), 169.2 (C-5'), 157.0 (C-1'), 156.5 (C-4a'), 155.7 (C-4a), 153.9 (C-5a), 153.4 (C-1), 150.9 (C-5a'), 141.7 (C-3/3') 141.0 (C-3/3'), 131.8 (C-2), 131.5 (C-4), 131.3 (C-4'), 130.2 (C-2'), 125.1 (C-6'), 125.0 (C-6), 107.8 (C-11'), 105.1 (C-8'), 91.4 (C-7a), 87.8–86.2 (C-9'/10'), 32.0 (C-12'), 22.3 (C-13'), 18.9 (C-7'), 8.7 ppm (C-7). ^31^P{^1^H} NMR (acetone-*d*_*6*_): δ –144.3 ppm (sept, ^*2*^ *J* = 708 Hz, PF_6_^−^). MS (ESI^+^): *m/z* 460.0785 [M – 2PF_6_]^2+^ (m_calc_ = 460.0769). EA calculated for C_38_H_43_Cl_2_F_12_IrN_4_P_2_Ru·0.9H_2_O: C 37.22%, H 3.68%, N 4.57%. Found: C 36.87%, H 3.72%, N 4.95%.

[Chlorido(η^5^-pentamethylcyclopentadienyl)iridium(III)](N,N'-(1,4-phenylene)(bis(1-(pyridin-2-yl)(methanimine)-*κ* ^2^N,N')[chlorido(η^6^-p-cymene)osmium(II)] hexafluorophosphate **2f**.

The synthesis was performed according to the general procedure using **1c** (163 mg, 0.23 mmol), 2-pyridine carboxaldehyde (22 µL, 0.23 mmol), [Os(cym)Cl_2_]_2_ (91 mg, 0.12 mmol) and NH_4_PF_6_ (753 mg, 4.6 mmol) to afford **2f** as a dark red powder (201 mg, 67%). m. p.: 147°C (clear point). ^1^H NMR acetone-*d*_*6*_): δ 9.64 (d, ^3^ *J* = 6 Hz, 1H, H-1'), 9.49–9.47 (m, 2H, H-5/5'), 9.25 (d, ^3^ *J* = 6 Hz, 1H, H-1), 8.59–8.54 (m, 2H, H-4/4'), 8.44 (td, ^3^ *J* = 8 Hz, ^3^ *J* = 1 Hz, 1H, H-3), 8.38 (m, 1H, H-3'), 8.16–8.12 (m, 2H, H-6'), 8.11–8.04 (m, 3H, H-2/6), 7.94–7.89 (m, 1H, H-2'), 6.50–6.44 (m, 1H, H-9'), 6.08–5.98 (m, 2H, H-8'/9'), 5.87 (dd, ^3^ *J* = 20.6 Hz, ^4^ *J* = 5.8 Hz, 1H, H8'), 2.63 (sept, ^3^ *J* = 7 Hz, 1H, H-10'), 2.39 (d, ^3^ *J* = 7.2 Hz, 3H, H-7'), 1.67–1.62 (m, 15H, H-7), 1.12–1.07 ppm (m, 6H, H-11'). ^13^C{^1^H} DEPT-Q (acetone-*d*_*6*_): δ 170.7 (C-5/5'), 170.1 (C-5/5'), 157.1 (C-4a'), 156.5 (C-4a) 156.7 (C-1'), 154.0 (C-5a), 153.4 (C-1), 151.0 (C-5a'), 141.7 (C-4), 141.1 (C-4'), 131.8 (C-2), 131.4 (C-3/3'), 131.1 (C-2'), 125.4 (C-6/6'), 125.15 (C-6/6'), 99.6 (C-11'), 99.4 (C-8'), 91.4 (C-7a), 79.6–76.7 (C-9'/10'), 32.2 (C-12'), 22.6 (C-13'), 19.0 (C-7'), 8.7 ppm (C-7). ^31^P{^1^H} NMR (acetone-*d*_*6*_): δ –144.3 ppm (sept, ^*2*^ *J* = 708 Hz, PF_6_^−^). MS (ESI^+^): *m/z* 505.1053 [M – 2PF_6_]^2+^ (m_calc_ = 505.1055). EA calculated for C_38_H_43_Cl_2_F_12_IrN_4_P_2_Os·0.6H_2_O: C 34.84%, H 3.41%, N 4.28%. Found: C 34.44%, H 3.29%, N 4.04%.

### 4.3 DMSO and Aqueous Stability Studies

Stability studies in DMSO were conducted for **2c** and **2f** by dissolving *ca*. 1 mg of the complex in DMSO-*d*_*6*_ (*ca*. 0.5 ml). ^1^H NMR spectra were recorded at *t* = 0, 2, 6, 24, 48, and 72 h.

The stability studies in aqueous solution were conducted by dissolving *ca*. 1 mg of **2c** or **2f** in DMSO-*d*_*6*_ (0.05 ml) and diluting it with D_2_O (0.45 ml) to form a 10% DMSO-*d*_*6*_/D_2_O solution. ^1^H NMR spectra were recorded at *t* = 0, 2, 6, 24, 48, and 72 h. The compounds were investigated *via* the same procedure in a solution of 100 mM NaCl in D_2_O (0.45 ml) added to a solution of *ca*. 1 mg of the complex in DMSO-*d*_*6*_ (0.05 ml). In addition, a solution of the hydrolyzed product was prepared by the addition of AgNO_3_ (2 eq) to a suspension of *ca*. 1 mg of the complex (1 eq) in 10% DMSO-*d*_*6*_/D_2_O. After vigorous shaking, the formed AgCl was removed by filtration and a ^1^H NMR spectrum of the filtrate was recorded.

### 4.4 Cell Cytotoxicity Studies

The antiproliferative activity of compounds **2a**–**2f** was investigated in HCT116, SW480, SiHa and NCI-H460 cells as described elsewhere (Movassaghi et al., 2018). In brief, the cells were grown in α-MEM supplemented with 5% fetal calf serum at 37°C in a humidified incubator with 5% CO_2_ after seeding them at 750 (HCT116, NCI-H460), 4,000 (SiHa) and 5,000 (SW480) cells per well in 96-well plates. The complexes were added to the plates in a series of 3-fold dilutions in 0.5% DMSO at the highest concentration for 72 h before the assay was terminated and the cells were stained with 0.4% sulforhodamine B (Sigma-Aldrich). The IC_50_ values were calculated with SigmaPlot 14.0 (Systat Software Inc.) using a three-parameter logistic sigmoidal dose-response curve between the calculated growth inhibition and the compound concentration. The presented IC_50_ values are the mean of at least three independent experiments, where 10 concentrations were tested in duplicate for each compound.

## Data Availability

The original contributions presented in the study are included in the article/Supplementary Material, further inquiries can be directed to the corresponding author.
