# Supplementary material for: Synthetic Strategy Towards Heterodimetallic Half-Sandwich Complexes Based on a Symmetric Ditopic Ligand
Source: Front Chem. 2021 Dec 3;9:786367. doi: 10.3389/fchem.2021.786367 (PMC8677676; doi:10.3389/fchem.2021.786367)
Supplement: Supplementary file 1 [file DataSheet1.PDF]

Supplementary Material

Table of Contents

XRD crystallographic data and structures

^{1}H and $^{13}C\{^{1}H\}$ DEPT-Q spectra

DMSO and aqueous stability

Supplementary Table 1.

	$[M - PF_6]^+$		$[M - 2PF_6]^{2+}$	
	m/z_{exp}	m/z_{calc}	m/z_{exp}	m/z_{calc}
**2a**	1063.1156	1063.1067	459.0743	459.0710
**2b**	975.0672	975.0604	415.0514	415.0478
**2c**	not observed	-	not observed	-
**2d**	1067.1216	1067.1251	461.0797	461.0802
**2e**	1065.1157	1065.1167	460.0788	460.0760
**2f**	1155.1695	1155.1720	505.1053	505.1036

Supplementary Table 2.

	**2a**·CH_3CN	**2d**·½C_7H_8	**2e**·½H_2O·½C_7H_8 [a]
**CCDC number**	2111155	2111156	2111157
**Formula**	$C_{38}H_{42}Cl_2N_4P_2F_{12}Os_{0.9}Ru_{1.1}$ · CH_3CN	$C_{38}H_{44}Cl_2N_4P_2F_{12}Ir_{1.07}Rh_{0.93}$ · ½ C_7H_8	$C_{38}H_{43}Cl_2N_4P_2F_{12}IrRu$ · ½ H_2O [+ ½ C_7H_8] [a]
**Formula weight (g mol^{-1})**	1239.00	1265.04	1218.88
**Temperature (K)**	107.0(5)	100.0(1)	107(5)
**Crystal system**	Triclinic	Triclinic	Triclinic
**Space group**	*P*-1	*P*-1	*P*-1
**Unit cell dimensions:**			
a **(Å)**	8.5634(2)	8.5895(1)	8.6944(1)
b **(Å)**	15.1882(3)	17.3380(2)	11.0734(1)
c **(Å)**	18.7245(4)	17.8657(1)	24.8992(2)
**α (°)**	111.435(2)	118.381(1)	101.892(1)
**β (°)**	92.189(2)	99.909(1)	96.460(1)
**γ (°)**	98.490(2)	90.558(1)	94.527(1)
**Volume (Å^3)**	2230.81(9)	2293.03(4)	2317.98(4)
**Z**	2	2	2
**Calculated density (mg m^{-3})**	1.845	1.832	1.746
**Absorption coefficient (mm^{-1})**	10.342	11.119	10.561
**Reflections collected**	26610	65885	68602
**Independent reflections**	8085	8293	8496
**Data / restraints / parameters**	8085 / 0 / 577	8293 / 4 / 593	8496 / 0 / 549
**Final R indices [I>2σ (I)]**	R1 = 0.0405, wR2 = 0.1034	R1 = 0.0283, wR2 = 0.0669	R1 = 0.0270, wR2 = 0.0664
**R indices (all data)**	R1 = 0.0455, wR2 = 0.1065	R1 = 0.0320, wR2 = 0.0691	R1 = 0.0290, wR2 = 0.0673
**Goodness-of-fit on F^2**	1.056	1.032	1.036
**Largest diff. peak and hole (eÅ^{-3})**	4.17 / -2.00	1.45 / -0.87	1.41 / -1.26

[a] Disordered toluene solvent not resolved; molecule was modelled with BYPASS solvent mask in final refinement.

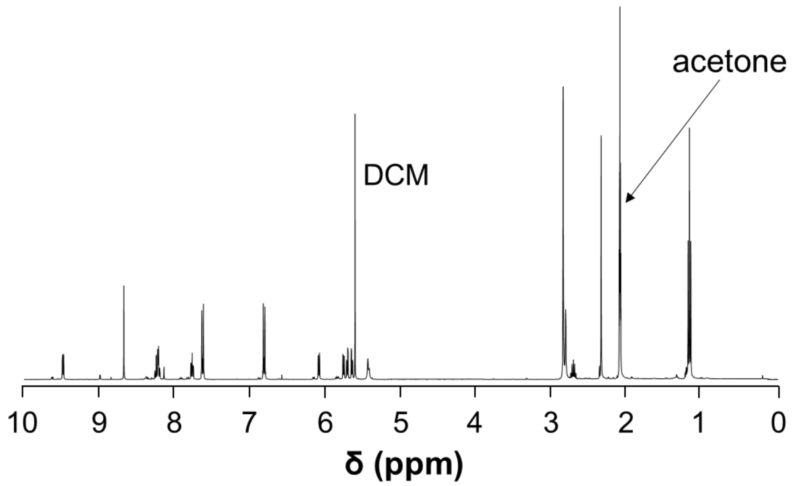

Supplementary Figure 1. ^{1}H NMR spectrum of **1a**.

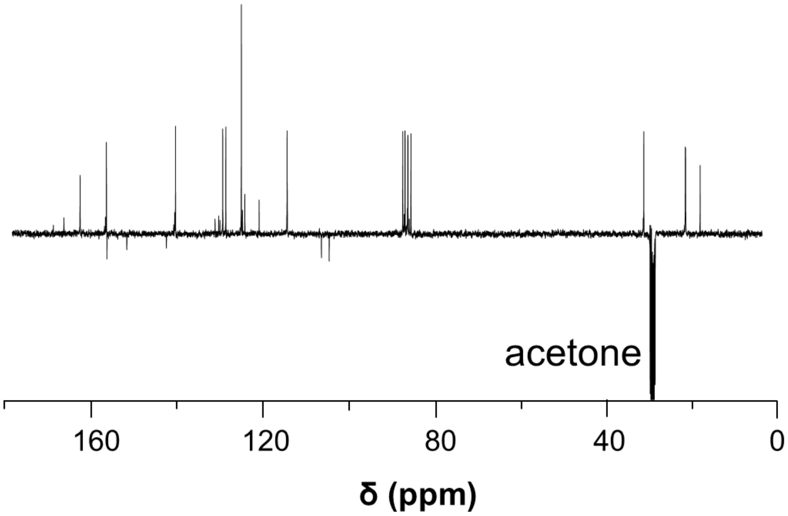

Supplementary Figure 2. $^{13}C\{^{1}H\}$ DEPT-Q NMR spectrum of **1a**.

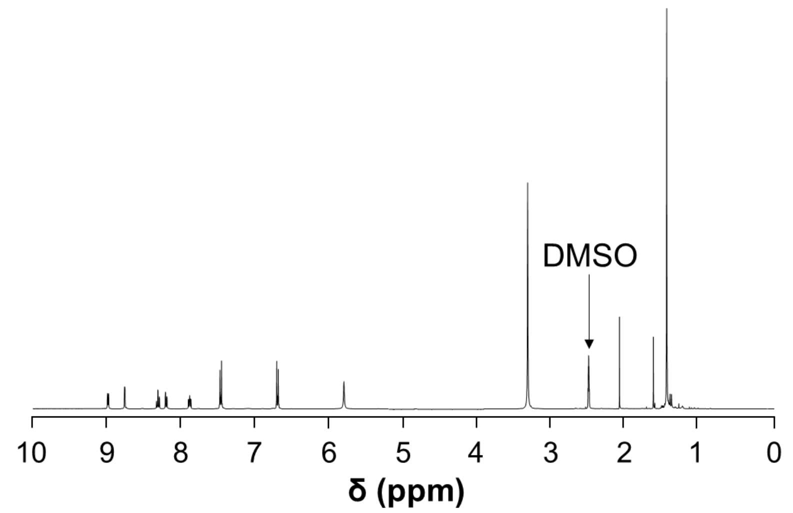

Supplementary Figure 3. ^{1}H NMR spectrum of **1b**.

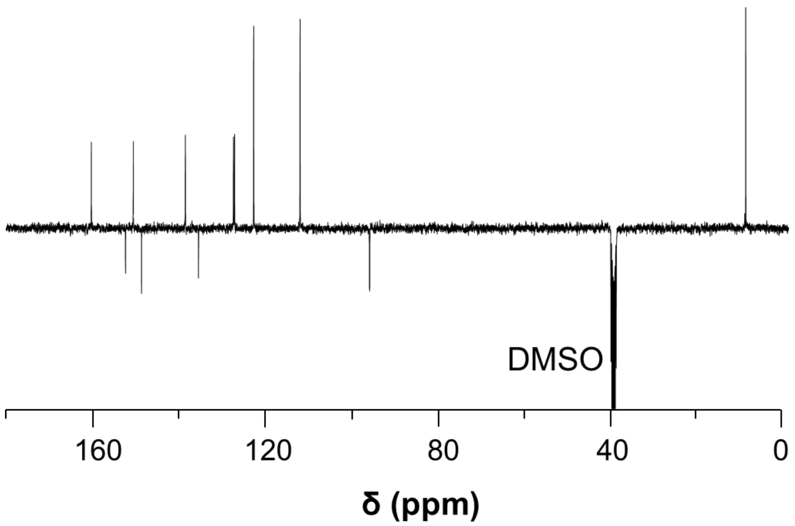

Supplementary Figure 4. $^{13}C\{^{1}H\}$ DEPT-Q NMR spectrum of **1b**.

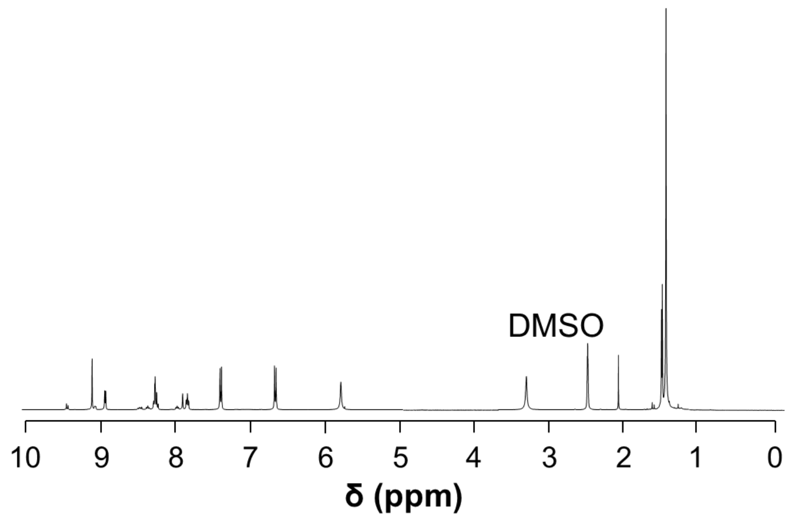

Supplementary Figure 5. ^{1}H NMR spectrum of **1c**.

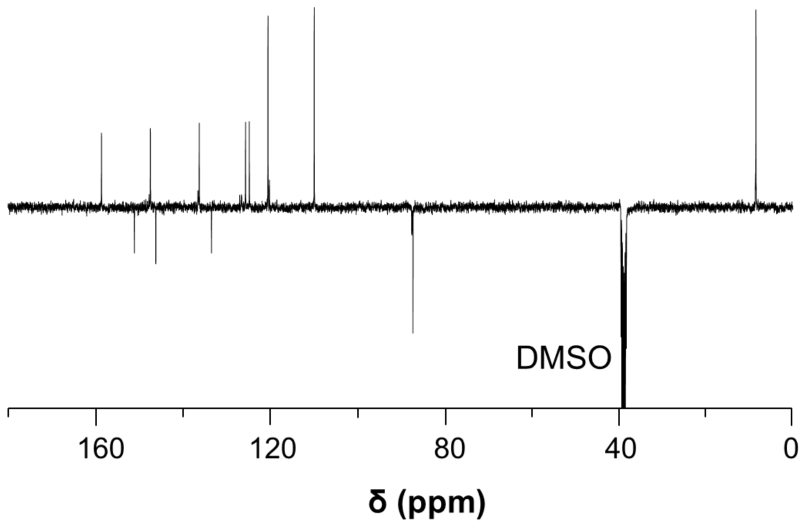

Supplementary Figure 6. $^{13}C\{^{1}H\}$ DEPT-Q NMR spectrum of **1c**.

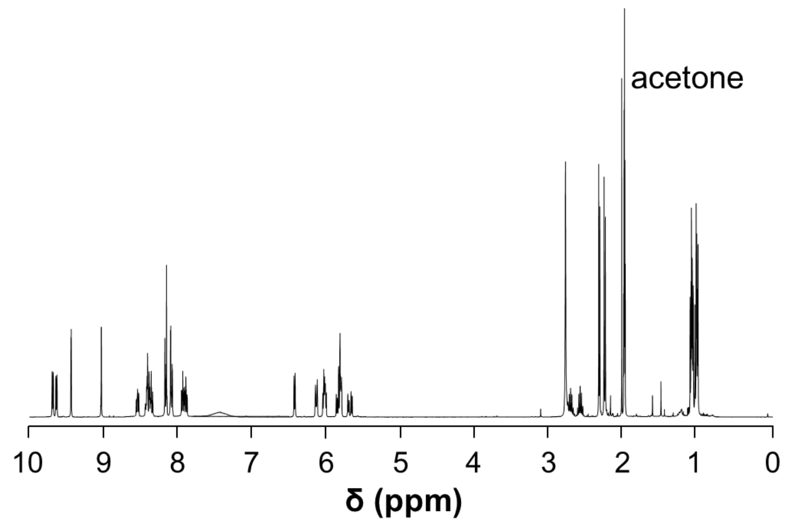

Supplementary Figure 7. ^{1}H NMR spectrum of **2a**.

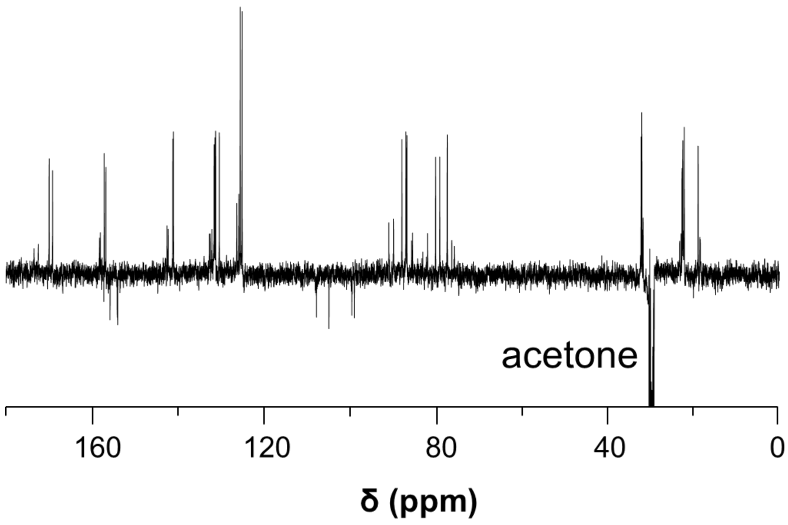

Supplementary Figure 8. $^{13}C\{^{1}H\}$ DEPT-Q NMR spectrum of **2a**.

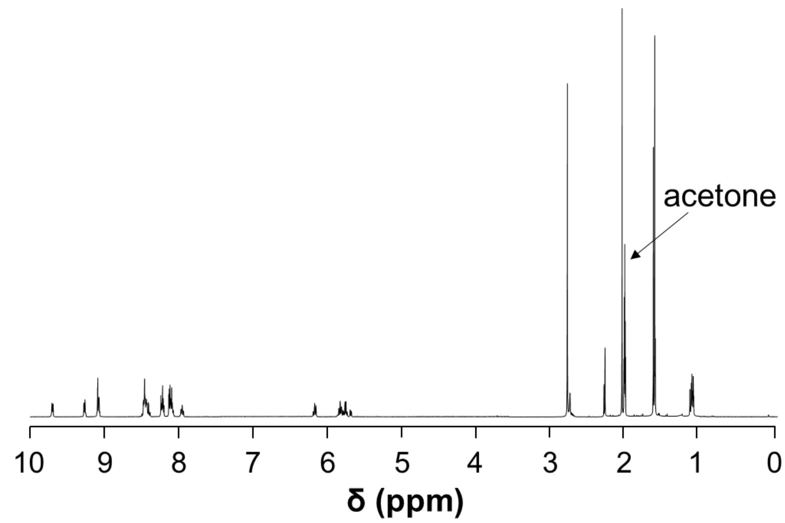

Supplementary Figure 9. ^{1}H NMR spectrum of **2b**.

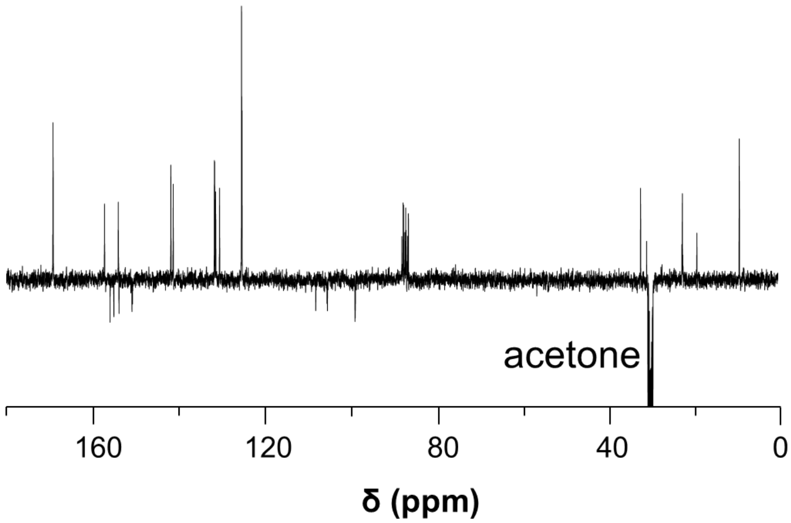

Supplementary Figure 10. ^{13}C{^{1}H} DEPT-Q NMR spectrum of **2b**.

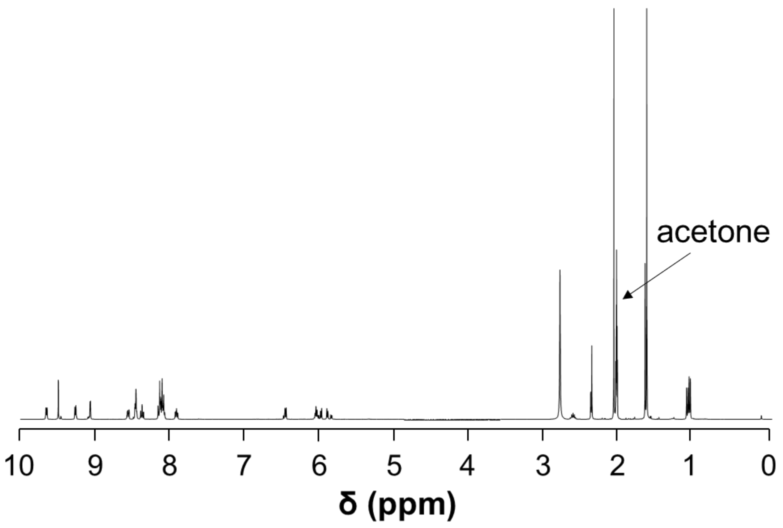

Supplementary Figure 11. ^{1}H NMR spectrum of **2c**.

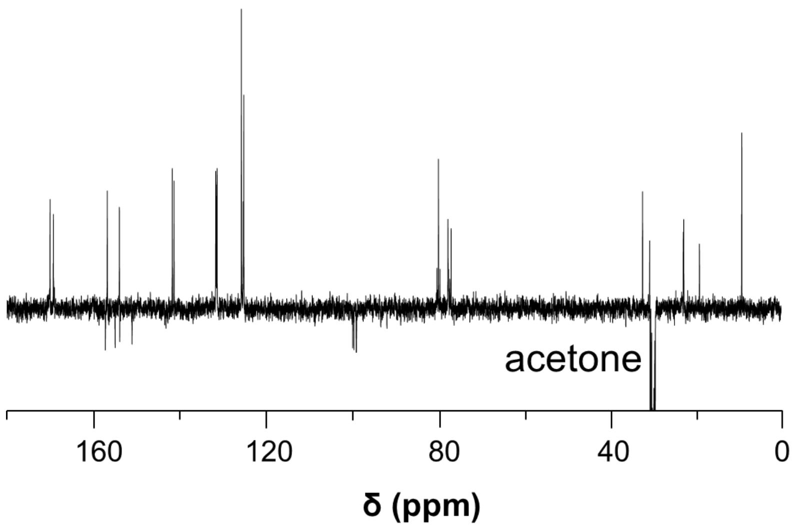

Supplementary Figure 12. $^{13}C\{^{1}H\}$ DEPT-Q NMR spectrum of **2c**.

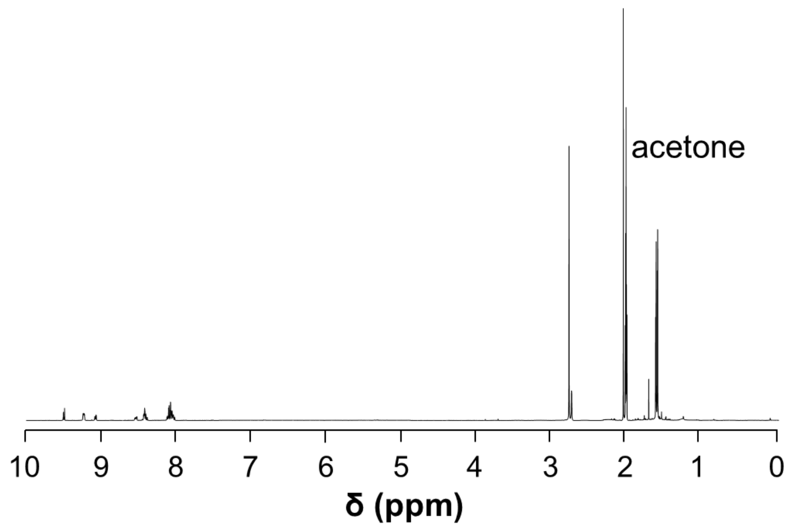

Supplementary Figure 13. ^{1}H NMR spectrum of **2d**.

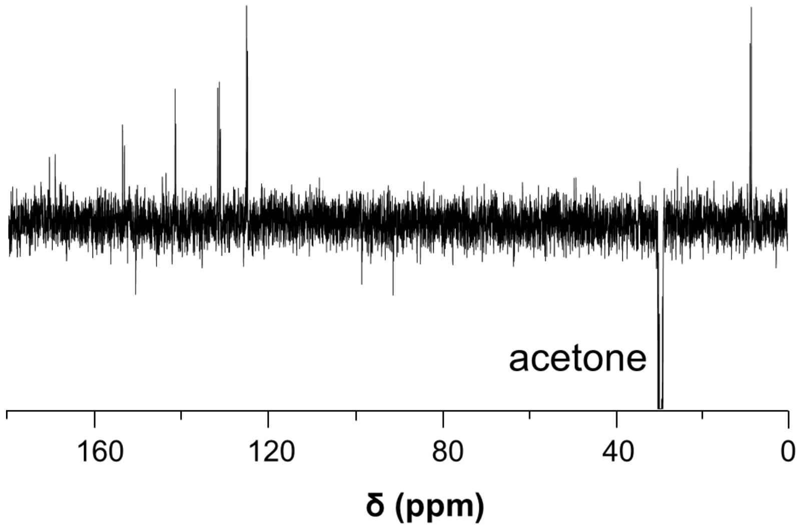

Supplementary Figure 14. $^{13}C\{^{1}H\}$ DEPT-Q NMR spectrum of **2d**.

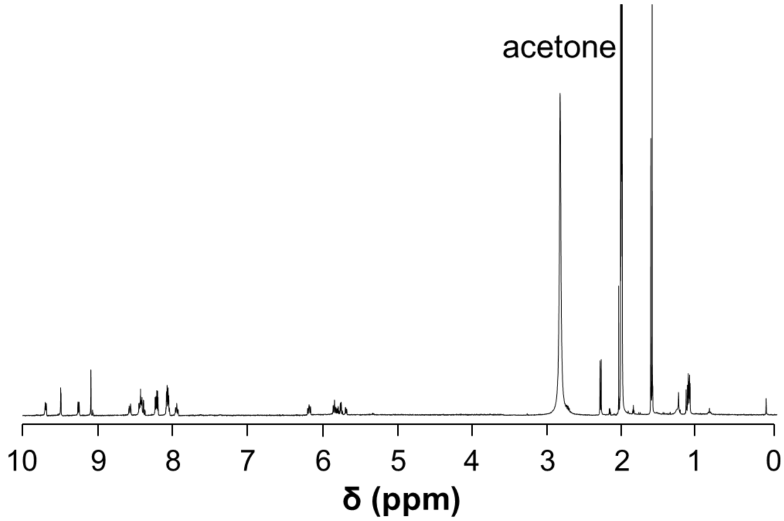

Supplementary Figure 15. ^{1}H NMR spectrum of **2e**.

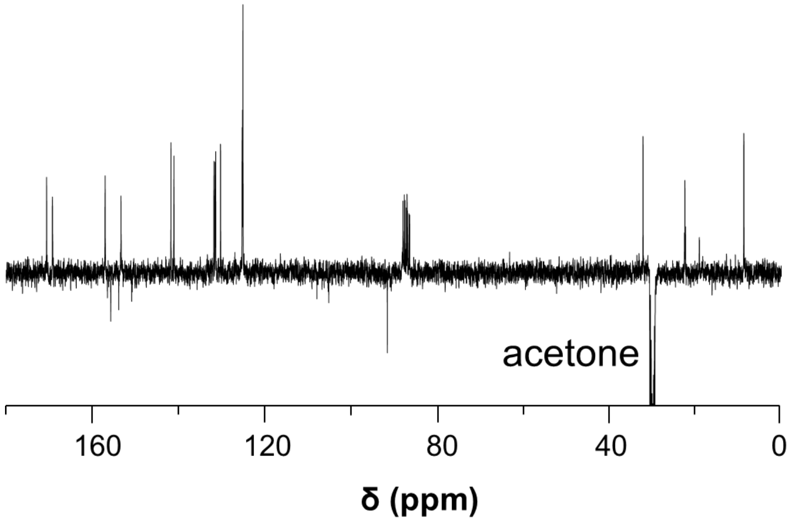

Supplementary Figure 16. $^{13}C\{^{1}H\}$ DEPT-Q NMR spectrum of **2e**.

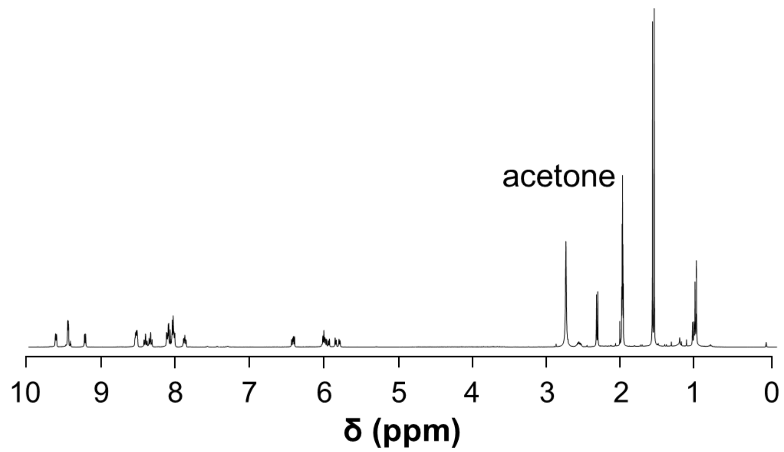

Supplementary Figure 17. ^{1}H NMR spectrum of **2f**.

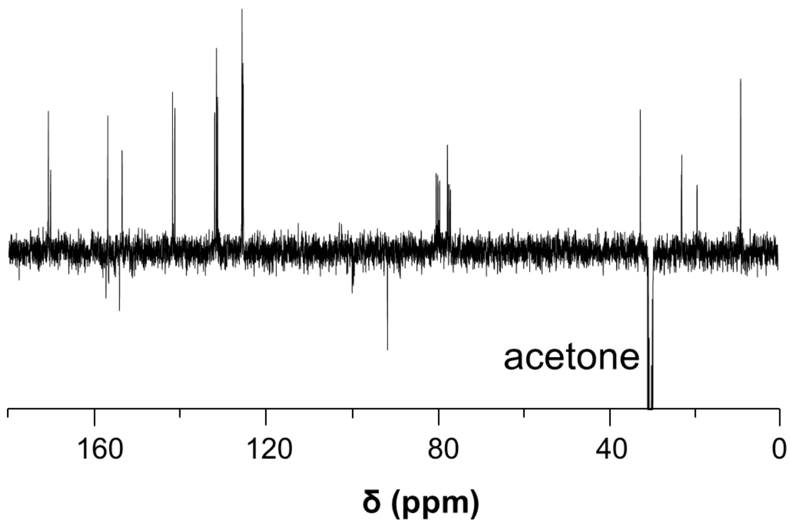

Supplementary Figure 18. ^{13}C{^{1}H} DEPT-Q NMR spectrum of **2f**.

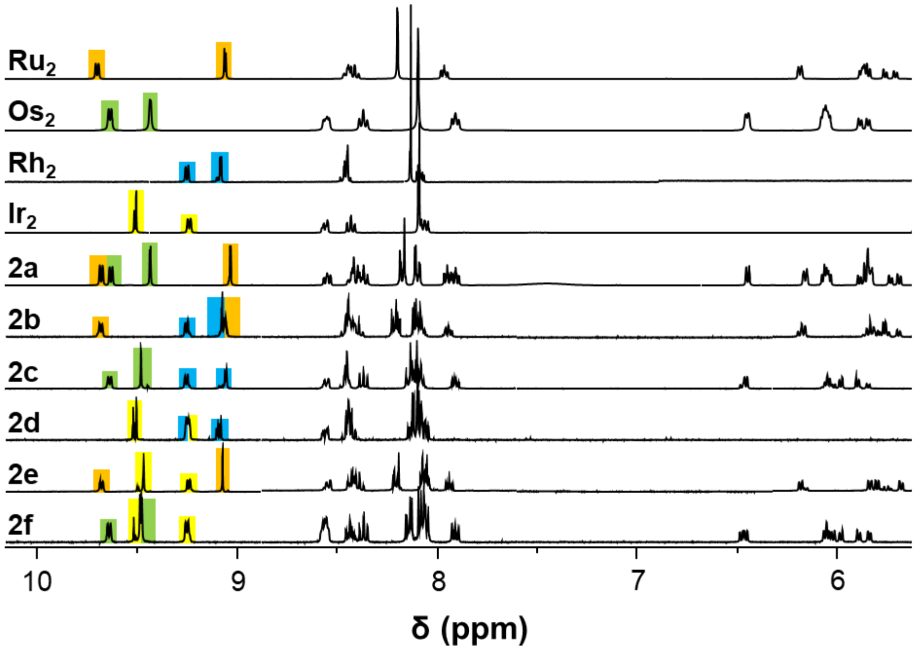

Supplementary Figure 19. Comparison of the ^{1}H NMR spectra of the homodimetallic **Ru2**, **Os2**, **Rh2** and **Ir2** with those of the heterodimetallic complexes **2a**–**2f** recorded in acetone-d$_6$.

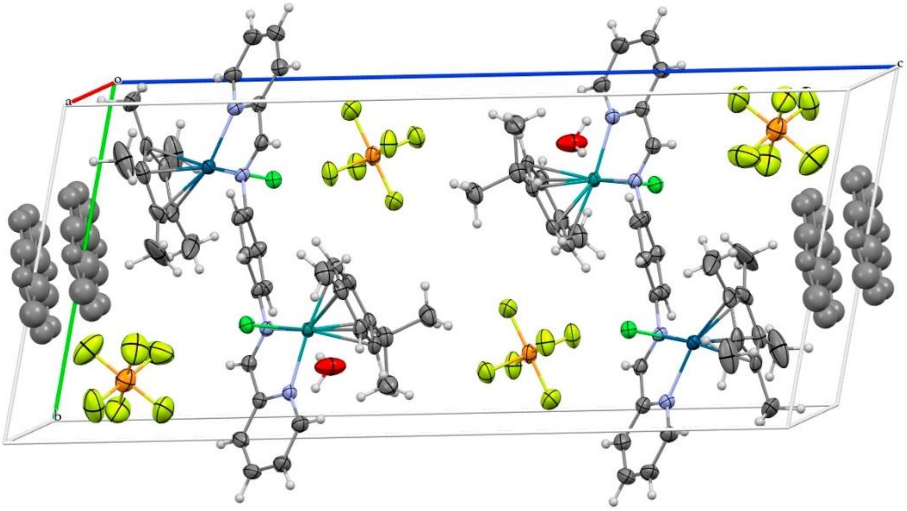

Supplementary Figure 20. Crystal structure of **2e**, including the strongly disordered toluene molecules π-stacked between two Cp* rings of adjacent complexes; the toluene molecule was not included in the final refinement as summarized in Table S2.

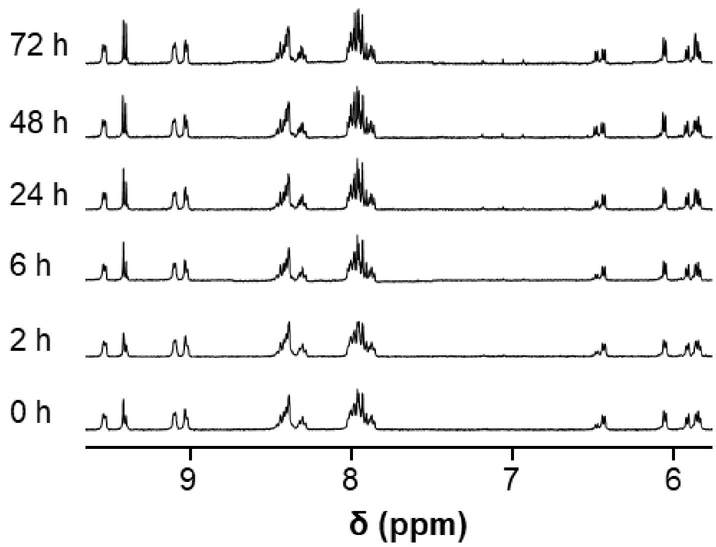

Supplementary Figure 21. ^{1}H NMR spectra of complex **2c** in DMSO-d_6 over a period of 72 h.

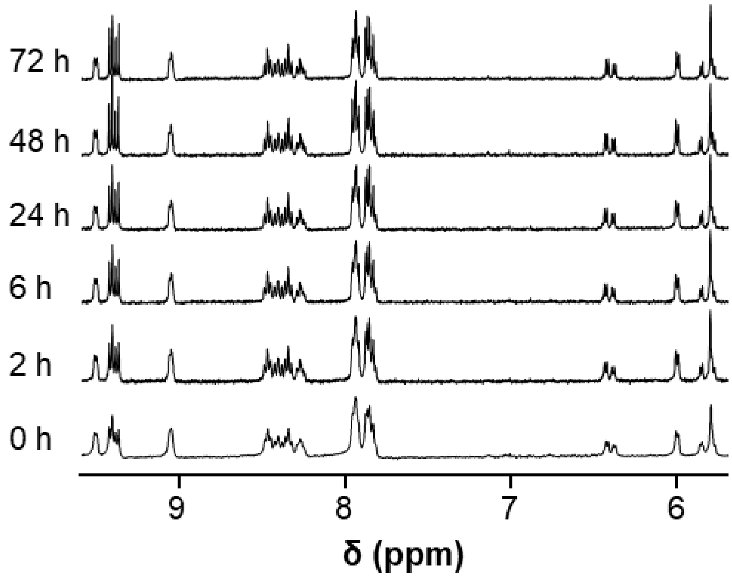

Supplementary Figure 22. ^{1}H NMR spectra of complex **2f** in DMSO-d_6 recorded over a period of 72 h.

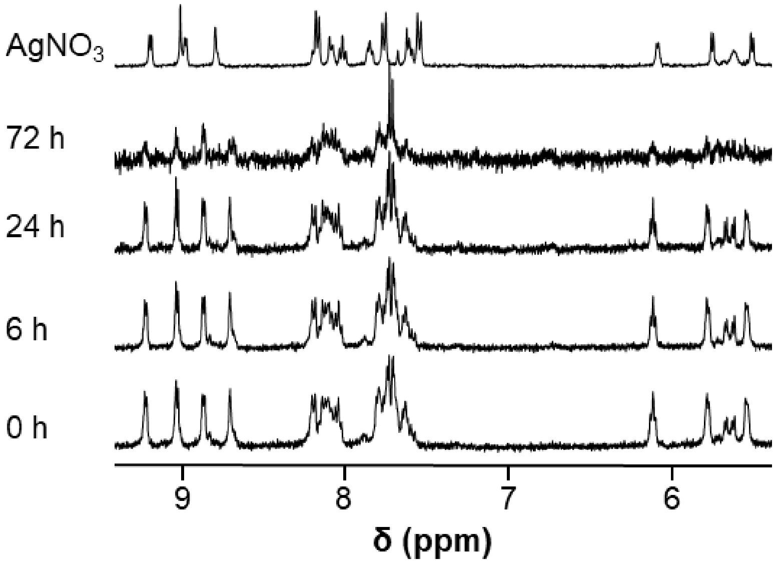

Supplementary Figure 23. ^{1}H NMR spectra of complex **2c** recorded in 10% DMSO-d_6/D_2O over a period of 72 h as well as after addition of $AgNO_3$ (2 equiv.).
